# MicroRNA Expression Profile in Human Macrophages in Response to *Leishmania major* Infection

**DOI:** 10.1371/journal.pntd.0002478

**Published:** 2013-10-03

**Authors:** Julien Lemaire, Ghada Mkannez, Fatma Z. Guerfali, Cindy Gustin, Hanène Attia, Rabiaa M. Sghaier, Sysco-Consortium, Koussay Dellagi, Dhafer Laouini, Patricia Renard

**Affiliations:** 1 Laboratory of Biochemistry and Cellular Biology (URBC), NARILIS-University of Namur, Namur, Belgium; 2 Institut Pasteur de Tunis, LR11IPT02, Laboratory of Transmission, Control and Immunobiology of Infections (LTCII), Tunis-Belvédère, Tunisia; 3 Université Tunis El Manar, Tunis, Tunisia; 4 Institut de Recherche pour le Développement (IRD) et Centre de Recherche et de Veille sur les Maladies Emergentes dans l'Océan Indien (CRVOI), Sainte Clotilde, Reunion Island, France; National Institute of Allergy and Infectious Diseases, United States of America

## Abstract

**Background:**

*Leishmania* (*L.*) are intracellular protozoan parasites able to survive and replicate in the hostile phagolysosomal environment of infected macrophages. They cause leishmaniasis, a heterogeneous group of worldwide-distributed affections, representing a paradigm of neglected diseases that are mainly embedded in impoverished populations. To establish successful infection and ensure their own survival, *Leishmania* have developed sophisticated strategies to subvert the host macrophage responses. Despite a wealth of gained crucial information, these strategies still remain poorly understood. MicroRNAs (miRNAs), an evolutionarily conserved class of endogenous 22-nucleotide non-coding RNAs, are described to participate in the regulation of almost every cellular process investigated so far. They regulate the expression of target genes both at the levels of mRNA stability and translation; changes in their expression have a profound effect on their target transcripts.

**Methodology/Principal Findings:**

We report in this study a comprehensive analysis of miRNA expression profiles in *L. major*-infected human primary macrophages of three healthy donors assessed at different time-points post-infection (three to 24 h). We show that expression of 64 out of 365 analyzed miRNAs was consistently deregulated upon infection with the same trends in all donors. Among these, several are known to be induced by TLR-dependent responses. GO enrichment analysis of experimentally validated miRNA-targeted genes revealed that several pathways and molecular functions were disturbed upon parasite infection. Finally, following parasite infection, miR-210 abundance was enhanced in HIF-1α-dependent manner, though it did not contribute to inhibiting anti-apoptotic pathways through pro-apoptotic caspase-3 regulation.

**Conclusions/Significance:**

Our data suggest that alteration in miRNA levels likely plays an important role in regulating macrophage functions following *L. major* infection. These results could contribute to better understanding of the dynamics of gene expression in host cells during leishmaniasis.

## Author Summary

*Leishmania* parasites belong to different species, each one characterized by specific vectors and reservoirs, and causes cutaneous or visceral disease(s) of variable clinical presentation and severity. In its mammalian host, the parasite is an obligate intracellular pathogen infecting the monocyte/macrophage lineage. *Leishmania* have developed ambiguous relationships with macrophages. Indeed, these cells are the shelter of invading parasites, where they will grow and eventually will reside in a silent state for life. But macrophages are also the cells that participate, through the induction of several pro-inflammatory mediators and antigen presentation, to shape the host immune response and ultimately kill the invader. To subvert these anti-parasite responses, *Leishmania* manipulate the host machinery for their own differentiation and survival. We aimed to evaluate the impact of *L. major* (the causative agent of zoonotic cutaneous leishmaniasis) infection on deregulation of non-coding miRNAs, a class of important regulators of gene expression. Our results revealed the implication of several miRNAs on macrophage fate upon parasite infection through regulation of different pathways, including cell death. Our findings provided a new insight for understanding mechanisms governing this miRNA deregulation by parasite infection and will help to provide clues for the development of control strategies for this disease.

## Introduction

The protozoan parasite *Leishmania* (*L.*) causes a heterogeneous group of tropical and subtropical neglected diseases known as leishmaniasis, with symptoms ranging from cutaneous lesions to fatal visceral leishmaniasis [1].

*Leishmania* parasites are obligate intracellular pathogens of their mammalian hosts. To establish infection, the flagellated metacyclic promastigote form is inoculated into host tissue through the bite of a female sandfly. It then electively invades macrophages where it differentiates into the highly replicative amastigote form whilst avoiding and/or subverting anti-parasitic responses [2], [3].

As dual actors (i.e., being the host cells that allow parasite replication as well as the effector cells that are responsible for parasite killing), macrophages play, beside neutrophils, a central role for host resistance or susceptibility to *Leishmania* infection [4], [5].

As successful intracellular parasites, *Leishmania* have developed a range of sophisticated strategies to subvert and/or suppress leishmanicidal activities of macrophages and overcome the host innate immunity. Indeed, *Leishmania* parasites inhibit, upon infection, antigen presentation [6], alter expression of co-stimulatory molecules [7], disturb signaling pathways and transcription factors activities [3], [8], [9], [10], affect cytokine [11] and chemokine [12] profiles and modulate metabolic pathways [13].

To safely ensure their differentiation into amastigotes, the replicative intracellular form of the parasite, *Leishmania* inhibit macrophage apoptosis [14] through complete remodeling of host apoptotic and anti-apoptotic transcripts [15] and more specifically, through the repression of mitochondrial release of cytochrome c [16] or activation of PI3K/Akt signaling [17].

An exciting pattern of gene regulation in plants and animals has recently emerged with the discovery of mammalian microRNAs (miRNAs), which are a class of endogenous non-coding small RNAs that regulate target mRNAs. Corresponding to approximately 1–2% of the known eukaryotic genomes, miRNAs are now considered as master regulators of gene expression for at least 30% of human genes [18]. In addition, computational predictions suggest that each miRNA can target 100 or more transcripts and that a single mRNA may be regulated by multiple miRNA species [19], [20]. Functional studies indicate that miRNAs take part in the regulation of every cellular process investigated so far and are involved in many pathologic processes and diseases.

During infection, changes in the host cell miRNA profile may either indicate a cell defense mechanism or a subversion strategy developed by the pathogen. Several classes of pathogens including viruses [21], [22], bacteria [23], [24] and apicomplexan parasites [25] like the intracellular protozoan parasite *Toxoplasma gondii* [26] can manipulate the miRNA network of infected host cells. Hence, we hypothesized that *Leishmania* might also alter macrophage host miRNA expression profile to convert the harsh phagolysosomal environment to a state suitable for its own survival and persistence.

We investigated whether *L. major*, the causative agent of zoonotic cutaneous leishmaniasis, drives changes in the miRNA-levels upon macrophage infection. For that purpose, we profiled a set of 365 miRNAs at time points 3, 6, 12, and 24 h post-infection and showed that *L. major* infection of human primary macrophages modifies the expression of about 20% of them. Interestingly, several of these differentially expressed regulatory molecules are known to be LPS- and/or TLR ligand-induced miRNAs [27]. As far as we know, this is the first study providing a valuable framework on human macrophage miRNA profile upon *L. major* infection that might be useful to identify new targets for anti-parasitic therapy.

## Materials and Methods

### Ethics statement

The study protocol, consent forms and procedures were reviewed and approved by the Institut Pasteur de Tunis Ethical Review Board of. Healthy volunteer individuals provided written informed consent for the collection of blood and subsequent analysis.

### Macrophage differentiation and infection

Healthy volunteer blood donors were selected as negative for any recent infection and had no history of leishmaniasis. Their peripheral blood mononuclear cells (PBMC) did not proliferate *in vitro* on exposure to Soluble *Leishmania* Antigens and they were not taking any medication at the time of the study.

PBMC were isolated from cytapheresis leukopacks using Ficoll-Paque (Pharmacia, Uppsala, Sweden) density gradient centrifugation. Cells were washed and incubated at 10^7^ cells/mL in RPMI 1640 medium supplemented with 2 mM L-glutamine, 100 U/mL penicillin, 100 µg/mL streptomycin and 5% heat inactivated fetal calf serum. Monocytes were purified by fibronectin-mediated adhesion using gelatin (Sigma) and autologous heat inactivated serum substratum [28]. Cell purity was assessed by flow cytometry (FACSVantage; Becton Dickinson, Sunnyvale, CA) using directly conjugated anti-CD3, anti-CD19 and anti-CD14 antibodies (Becton-Dickinson, San Jose, CA) and was routinely greater than 85% of CD14^+^ cells. Macrophages were derived from monocytes cultured for 8 days in 6- or 24-well tissue-culture plates, at 37°C, 5% CO_2_ in endotoxin-free RPMI 1640 medium supplemented with 5% heat-inactivated normal human AB serum and 10% heat-inactivated fetal calf serum (HyClone Laboratories, Logan, UT), 100 U/mL penicillin, 100 µg/mL streptomycin, 2 mM L-glutamine at 2×10^6^ cells/mL.

Macrophages Derived Monocytes (MDM) were then exposed to metacyclic promastigotes of *L. major* (MHOM/TN/95/GLC94 strain). Parasites at their infective stage were collected by density gradient centrifugation from stationary culture (6–7 days old) [29]. MDM infection was conducted at a ratio of 5 parasites per cell for 3, 6, 12 and 24 hours (IF: infected) and then harvested for analysis. When available, MDM were also cultured in the presence of the same ratio of latex beads as phagocytosis control (Sigma, St. Louis, MO). Non-infected MDM were collected at the same time points and used as controls (NI: non infected).

### Macrophage transfection

MDM were transfected twice by using the HiPerFect Transfection Reagent (Qiagen), following the procedure described by Hulten [30]. As a single transfection protocol gave low efficiency (Lemaire, personal communication), a double transfection, as recommended by the manufacturer and other studies, was used to improve knockdown efficiency. Briefly, 20 mM of siRNA were mixed with 12 µl of HiPerFect reagent and incubated for 10 min at room temperature. The mix was resuspended in 200 µl of endotoxin-free RPMI 1640 medium with antibiotics (100 U/mL penicillin, 100 µg/mL streptomycin) and added to the cells, previously washed twice with PBS. Cells were incubated at 37°C for 6 h before adding 500 µl of RPMI 1640 medium with antibiotics supplemented with 10% heat-inactivated fetal calf serum. Cells were incubated at 37°C and the transfection performed once again after the 24 h of incubation. The siRNA used are anti-miR-210 miRNA inhibitor (AM10516, Ambion), anti-HIF-1α siRNA (SI04249308, Qiagen) or negative control siRNA (1027280, Qiagen). In order to verify that transfected macrophages were not activated by siRNA and remain at rest, TNF-α, IL-6, IL-8 and iNOS mRNA and/or protein levels but also nitric oxide amounts [31] were assessed. This was done using real-time RT-PCR, ELISA and Griess assays respectively, in control siRNA transfected cells, compared to non transfected cells.

### RNA isolation and quantitative reverse transcription-PCR analysis

#### RNA isolation

Cells were collected by centrifugation at the indicated time points by centrifugation, washed to remove extracellular parasites, homogenized by Trizol reagent (Gibco BRL) and frozen at −80°C until RNA extraction. The RNA from each time point was extracted using miRNA Qiagen kit according the manufacturer's instructions. RNA were quantified using NanoDrop ND-1000 micro-spectrophotometer, their integrity assessed using Agilent-2100 Bioanalyzer and stored at −80°C.

#### MiRNA expression profiling

The expression of 365 human miRNA species was analyzed by real-time PCR using microfluidic cards (TaqMan Human MicroRNA Array v1.0, Applied Biosystem) following the recommendations of the manufacturer. The abundance of each miRNA was normalized to the geometric average of the 2 endogenous controls, RNU44 and RNU48, according to [32], generating ΔCt values. ΔΔCt were calculated as the difference between infected and non-infected ΔCt. The results are expressed in fold change, corresponding to 2^−ΔΔCt^. Values below the background or undetectable are indicated as ND (not detectable).

#### Real-time PCR for miRNA array validation and chemokine transcript expression

RNA contained in 10 to 50 ng (depending on the targeted miRNA) was reverse transcribed using Taqman microRNA reverse transcription kit (Applied Biosystems, Foster City, CA, USA) and specific primers for each miRNA (miRBase, Applied Biosystems), following the recommendations of the manufacturer. Amplification reaction assays were performed with Taqman Universal PCR Master mix (Applied Biosystems) with specific primers (Applied Biosystems). RNU44 was used as the endogenous control for normalization and miRNA expression level was quantified using the 2^−ΔΔCt^ method. Chemokine mRNA measurements on material obtained from the same three initial donors were performed using SYBR Green I Universal PCR MasterMix (PE Applied Biosystems). Chemokine specific and housekeeping gene (β2-M, HPRT1 and GADPH) primers were obtained from SABiosciences. Results were expressed using the 2^−ΔΔCt^ method.

### Western blot

#### Total protein extraction

At indicated times, MDM were washed in cold PBS (2 mL/well) by centrifuging the culture plates at 450 g, 10 min, 4°C and cells were scrapped in cold PBS (500 µL/well) and transferred to microtubes. An additional volume of cold PBS (500 µL/well) was used to harvest cells remaining in the wells and was added to the same micro-tubes. To pellet cells, micro-tubes were centrifuged at 220 g, (10 min, 4°C). After removing supernatant, an additional 3–5 min centrifugation step was used to completely dry the pellet. 100 µL of freshly thawed lysis buffer (Urea 7 M, Thiourea 2 M, CHAPS 2%, Tris HCl pH 8.8 30 mM, Protease Inhibitor 4%) was added to each pellet and tubes were kept at −80°C until use. Protein concentration was assayed using the Bradford protein assay (Bio-Rad).

#### Western blot

Equal amounts of total proteins (10 µg) were separated by SDS-PAGE on 10% acrylamide gels and transferred to a PVDF membrane. After blocking in TBS containing 0,1% Tween 20 and 2% milk (GE Healthcare Biosciences), the blots were probed with anti-caspase 3 antibody (#9662, Cell Signaling; dilution 1∶1,000). Chemiluminescent detection was performed using horseradish peroxidase-conjugated secondary antibodies and membranes were revealed with ECL (GE Healthcare Biosciences). Loading controls were checked using an antibody to HSP27 (sc-1048, Santa Cruz; dilution 1∶5,000). The first lane of the blot contained 10 µg of *Leishmania* lysate, to ensure that the antibodies used in the experiment did not cross-react with *Leishmania* proteins. Data shown are representative of three independent experiments conducted on MDM derived from three different healthy donors.

### Bioinformatics analyses

Analysis of miRNA differentially regulated after *L. major* infection was carried out for each experimental time point separately using the MultiExperiment Viewer (MeV) v4.7.1 from the TM4 software package [33] available as open-source software at http://www.tm4.org/mev.html.

Hierarchical Clustering was performed using the Euclidean distance metric with complete linkage option.

For miRNA target identification, we used the miRWalk comprehensive database that provides information on human miRNAs experimentally validated binding sites target genes [34] updated on 15^th^ March 2011.

InnateDB database [35] was used to classify all miRNA targets according to their associated GO terms using the hyper-geometric test and the Benjamini Hochberg correction method (default parameters). An enrichment analysis was performed using the BINGO plugIn [36] of Cytoscape [37] v2.8.3 [38], based on the GO terms revealed by the up- or down-regulated miRNA targets at each time point. We used the hyper-geometric test and the Benjamini Hochberg FDR correction method, and a 0.001 significance level due to the high proportion of associated GO terms. We finally used TransmiR database (updated on 19^th^ March 2012) for transcription factors (TFs) regulating miRNA transcription [39] to identify experimentally validated TFs that are upstream of deregulated miRNAs.

### Statistical analyses

The statistical significance of the quantitative differences between the different sample groups was determined by application of Student's two-tailed t test. P values of <0.05 were considered statistically significant.

## Results

### Profiling miRNA expression during *L. major* infection time course

To identify miRNAs for which expression is altered upon *L. major* infection of human macrophages, we incubated MDM from 3 healthy donors with metacyclic parasites. Macrophages were infected for 3, 6, 12 or 24 h and their RNA extracted for miRNA array assay. Percentage of infected cells and parasite load in all donors were microscopically assessed and showed consistent rates of infection (57% and 82% of infected cells; 5,6 and 6,2 parasites/infected cell at 12 and 24 h post-infection respectively).

RNA of non-infected MDM extracted at same time points were used as controls. Among the 365 human miRNAs assessed by Taqman real-time PCR (Table S1), 214 were either undetectable or below the background (ND); expression of the 151 remaining miRNAs was further analyzed further. Expecting a large inter-individual variability in miRNA expression as previously reported [40], [41], [42], we only selected miRNAs that showed consistent trends of deregulation (either up- or down-regulated) in the three donors with fixed cut-off values. According to this criterion, only 64 miRNAs had levels consistently modified by *L. major* infection (Table S1) and showed, using Principal Component Analysis observable on 3D graphs (Figure S1), the closest relationship and vicinity between the three donors compared to what was observed for the whole miRNA tested set.

**Figure 1:**
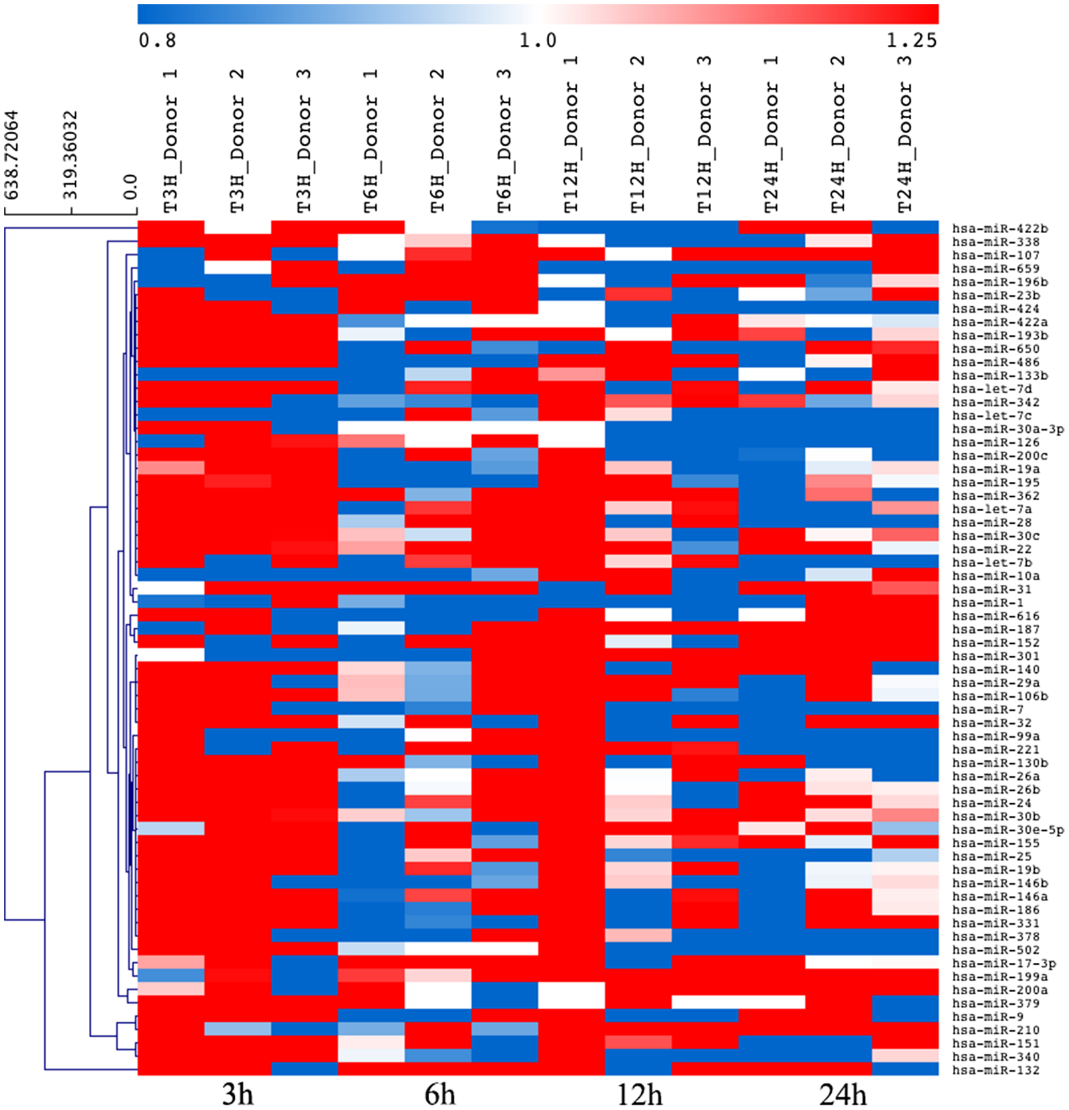
Hierarchical cluster analysis of deregulated miRNA expression in *L. major*-infected human primary macrophages. The miRNA expression values are presented using a red-white-blue color scheme, with red data points indicating higher expression than median values, white indicating expression equal to the median, and blue indicating lower expression than the median. MiRNAs were analyzed based on their expression before and at different time points, post-infection (3, 6, 12 and 24 h) of primary human macrophages from three healthy donors (D1, D2 and D3).

Hierarchical clustering of these 64 differentially regulated miRNAs is shown in Figure 1 and Figure S2 for the whole time-course and independent time-point course infection respectively. Figure 1 indicates that the proportion of up- or down-modulated miRNA is different for each time point. Hence, at 3 h post-infection, 31 miRNAs were up-regulated but only three were down-regulated. In contrast, at 6 h post-infection only five miRNAs were up-regulated and seven were down-regulated. Longer infection time showed eight and seven miRNAs up-modulated at 12 and 24 h respectively whereas three and 11 miRNAs were down-modulated at these time points. Finally, among the 64 miRNAs, four (miR-28, miR-331, miR-486 and miR-502) were differently deregulated at, at least, two different time points of infection. Control experiments using phagocytosis of inert beads did not generate similar alteration in miRNA expression Hence, the 64 miRNA, which levels were consistently modified by *L. major* infection are likely induced by parasite infection and unlikely reflecting only the early non-specific steps of phagocytic internalization (Table S1).

### QRT-PCR array validation

To validate the accuracy of array-generated data, a qRT-PCR validation study was carried out on nine selected miRNAs (miR- let7a, 26a, 26b, 130b, 132, 133b, 155, 199 and 210). This set was randomly selected from the 64 deregulated miRNAs and the number of qRT-PCR tested miRNAs was chosen merely proportionally to the deregulated numbers at each time point post-infection. In general, both data, generated by PCR-array or by qRT-PCR showed consistent results (up- or down-regulation) for most tested miRNAs in the three donors' MDM, though the magnitude of the response measured by the two approaches was different (Figure S3). The correlation coefficient between the mean values of the three donors (excepting two outliers values from the same donor measured by array PCR) for each miRNA measured by the two approaches was statistically significant (*r* = 0.78; *p* = 0.003).

### Identification and GO enrichment of differentially regulated miRNA targets

Since human miRNAs are able to regulate transcripts having only few nucleotides of complementarity, their potential to regulate large numbers of targets is obvious and amplifies the quantitative and qualitative consequences of miRNA modulation by *L. major* infection. In order to identify the transcripts that might be targeted by differentially regulated miRNA in infected macrophages, we used the validated module of miRWalk database, containing experimentally verified miR interaction information. The list of these targets is provided in Table S2.

According to this approach, genes belonging to critical cellular pathways were identified. Several noteworthy transcripts virtually targeted by deregulated miRNAs over the infection time course were highlighted. These highly targeted genes included AKT1 (v-akt murine thymoma viral oncogene homolog 1), BCL1 (B-cell CLL/lymphoma 1), BCL2, BCL2L11 (BCL2-like 11), EGFR (epidermal growth factor receptor), Jun, MCL1 (myeloid cell leukemia sequence 1), MYC (v-myc myelocytomatosis viral oncogene homolog), p53 and PTEN (phosphatase and tensin homolog), that belong to pro-, anti-apoptotic and proliferation pathways; IL-1β, IL-6, p50 NF-κB, p65 NF-κB, TLR-4 belonging to innate immune response pathways; NPC1 (Niemann-Pick disease, type C1) involved in intracellular cholesterol trafficking; PI3 (peptidase inhibitor 3) an anti-microbial peptide, Dicer1 and DROSHA involved in miRNA expression and CCND1 (cyclin D1) involved in the cell cycle.

**Figure 2:**
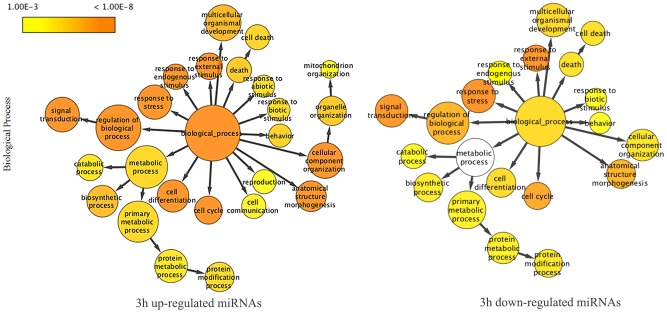
Biological processes deduced from analysis of deregulated miRNA-targets in *L. major*-infected human macrophages at 3 h post-infection. Yellow color gradient intensity correlates with up- or down-regulation levels. White nodes are not significantly overrepresented. The area of each node is proportional to the number of genes in the set annotated to the corresponding GO category. Interactions were visualized as a network using Cytoscape and BINGO plugin.

The target lists identified at each time point were then subjected to pathway analysis using the Cytoscape Plug-In BINGO after GO enrichment focusing on molecular functions, cellular components and biological processes. Several pathways have been highlighted during the infection time course. Assuming that there is theoretically a negative correlation between expression levels of miRNAs and their targets, we noticed several pathways and processes that could be either up- or down-regulated. Figure 2 shows affected up- and down-regulated biological processes of pathways targeted respectively by down- or up-modulated miRNAs in infected macrophages at 3 h. Other results (biological processes at other time points of infection and cellular components and molecular functions affected by parasite infection between 3 and 24 h) are represented in figures S4–S10.

Our results show that at 3 h post-infection several targets of up-modulated miRNAs are located in the plasma membrane of the infected macrophage whereas catalytic and enzyme regulator activities seem to be inhibited. Interestingly, several biological processes including cell communication and mitochondrion organization were also down-modulated early upon infection. GO enrichment also suggests that parasite infected macrophages down-regulate several processes including cellular movement, secretion, enzyme production or gene expression naturally induced through an abiotic stimulus.

At 6 h post-infection, our results predict an increase of catalytic and protein-binding activities associated with a more dynamic cell communication process. This continues at later times of course infection (12 and 24 h). Interestingly, this analysis predicted an inhibition of lipid binding molecular function, probably occurring within cytoplasmic membrane-bound vesicles concomitantly with an increase of enzyme regulator and receptor binding activities. Finally, at time when parasite infection appears to be well established (i.e., 24 h post-infection), symbiotic biological processes in response to stimulus seems inhibited. This might indicate either a shutdown of macrophage anti-parasitic responses or down-regulation of *Leishmania* key virulence gene activity, as a consequence of an already differentiated parasite invasion. Interestingly, several genes coding for parasite virulence factors are related to this biological process. Indeed, *L. major* inhibitor of serine peptidase 2 and 3 (ISP2 and ISP3), lipophosphoglycan 2 (LPG2) and leishmanolysin (gp63) are known as modulators of host immune response via regulation of its complement system, phagocytosis process and protein kinase-mediated and nitric oxide-mediated signal transduction. The expression of up to 27 human genes related to this process is known to be regulated by miRNAs. Interestingly, these genes include Natural resistance-associated macrophage protein 1 (SLC11A1), transportin 1 (TNPO1) and Transferrin receptor protein 1 (TFRC), among others.

We also predicted an enhanced activity in extracellular space and in signal transducers at 24 h.

It is worth noting that even if the identified pathways were either inhibited or enhanced in the same way at a given time point of infection, the number of involved targeted genes belonging to the same GO category might be different from one node to another.

Taken at the level of regulatory networks, this might reflect affected cellular processes and molecular functions in macrophages infected with *L. major* parasites following miRNA deregulation during the time course of infection.

### Negative correlation of expression between microRNAs and their mRNA targets

MiRNAs are known to regulate the expression of target genes both at the levels of mRNA translation and mRNA stability, leading to a negative correlation between expression levels of these master regulators and their target mRNAs. It was interesting to note that among the experimentally validated targeted transcripts of up-regulated miRNAs at 3 h, 6 h and 12 h (Table S2), several belong to the chemokine family (e.g., CCL2, CCL5, CXCL10, CXCL11 and CXCL12). These molecules might be inhibited by different miRNAs that were up-regulated upon *L. major* macrophage infection.

**Figure 3:**
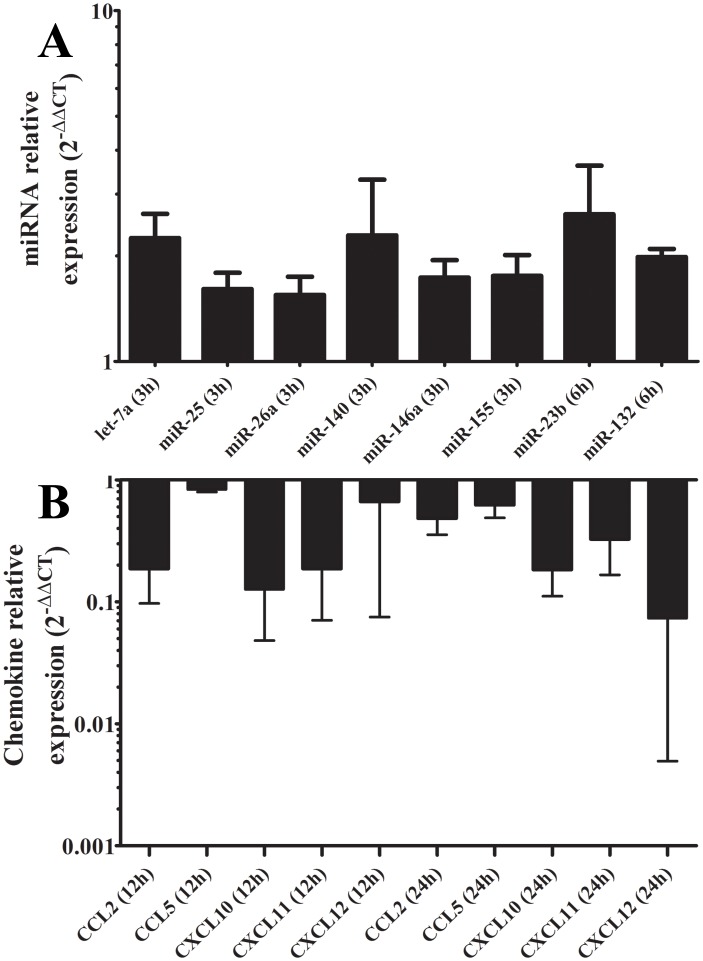
Negative correlation between expressions of an up-regulated set of miRNAs and their targeted chemokine transcripts. Expression means of let-7a, miR-25, miR-26a, miR-140, miR-146a and miR-155 at 3 h and miR-23b and miR-132 at 6 h post-infection of three healthy donors (D1, D2 and D3; panel A) is negatively correlated with CCL2, CCL5, CXCL10, CXCL11 and CXCL12 mRNA mean levels at 12 and 24 h post-infection (panel B) in *L. major*-infected human macrophages. Results were expressed using the 2^−ΔΔCt^ method.

In order to check if the mRNA expression of these chemokines was negatively correlated with the up-regulation of all the corresponding targeting miRNAs (i.e., let-7a, miR-25, miR-23b, miR-26a, miR-132, miR-140, miR-146a, miR-146b, miR-155 and miR-210) identified in Table S2, we measured their levels using qRT-PCR. Figure 3 shows the relative expression levels of let-7a, miR-25, miR-26a, miR-132, miR-140, miR-146a and miR-155 at 3 and 6 h (panel A), and of five chemokines of their predicted targets at 12 and 24 h (panel B).

Taken as a whole, and despite some individual variability in the measured levels between one donor to another as described elsewhere [40], [41], [42], these results clearly indicate that there is a statistically significant negative correlation (*r* = −0.27; *p* = 0.04) between expression levels of the selected up-regulated set of miRNAs and their corresponding chemokine targets (Figure S11). This negative correlation should be experimentally validated to indicate that the inhibition of chemokines-transcript levels is the direct result of regulation through expression of their targeting miRNAs.

### Identification of transcription factors regulating miRNA transcription

It is well known that the expression of miRNAs can be activated or repressed by TFs, which can serve as upstream regulators of miRNA expression. In order to indirectly identify TFs that are inhibited or activated by *L. major* infection, we used the TransmiR database listing experimentally validated TFs that are upstream regulators of miRNA expression (Table S3). Interestingly, several miRNAs were shown to be virtually activated, repressed or regulated by master transcription factors e.g., p50 NF-κB, EGR1, MYC, E2F1, PU.1, CREB-1, HIF-1α or p53. Among these TFs, at least CREB [43], HIF-1α [44] and p50 NF-κB [45] were previously shown to be modulated upon *Leishmania* infection.

### MiR -210 is partially controlled by HIF-1α activation but is not involved in down-regulation of pro-caspase-3 in parasite infected macrophages

Among the miRNAs for which levels were modulated during *L. major* infection, several were described as playing a possible role in apoptosis e.g., miR-210, [46] miR-22, miR-155 and miR-133b [47].

**Figure 4:**
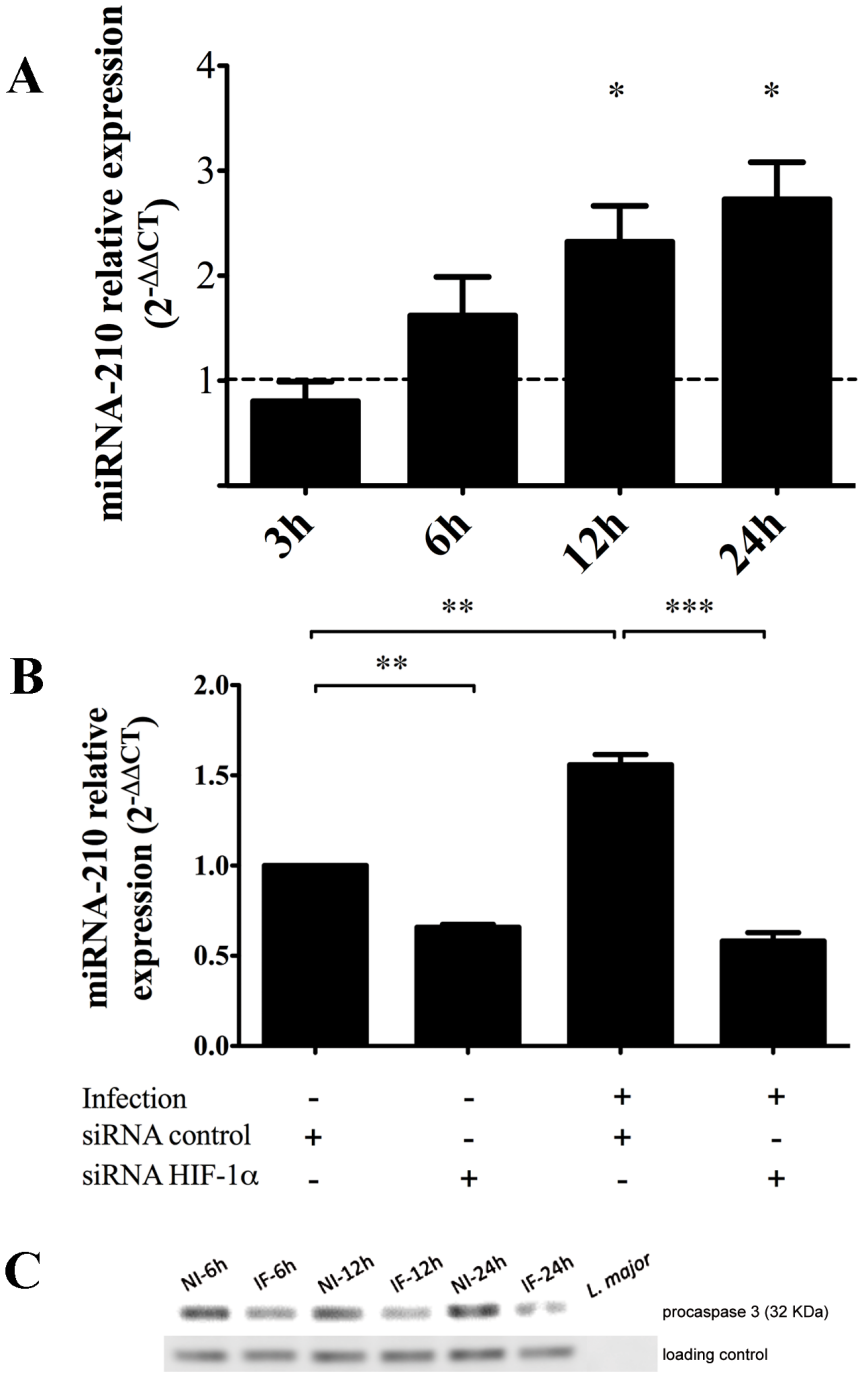
Time course of miRNA-210 and procaspase-3 expression levels in *L. major* infected human primary macrophages. MiR-210 expression at 3, 6, 12, and 24 h in infected cells relatively to non-infected cells (panel A), after siRNA-control or HIF-1α-silencing transfections in non-infected and infected cells (panel B). Results were expressed using the 2^−ΔΔCt^ method. Stars (*) are indicated when results are statistically significant from control. One star indicates a *p* value <0.05; two stars indicate a *p* value <0.01 and three stars indicate a *p* value <0.001. Panel C represents abundance of pro-caspase-3 protein levels in time course parasite-infected macrophages of healthy donors revealed by western blot analysis. HSP27 was used as loading control. Ten µg of *L. major* lysate (latest lane) was used a negative control to ensure that anti-procaspase-3 antibody does not cross-react with parasite proteins. Data are representative of three independent experiments conducted on MDM derived from two to three different healthy donors. NI indicates non-infected cells and IF indicates infected cells.

For this study, we focused on the role of one of these deregulated miRNA in infected macrophages, namely miR-210. Expression of miR-210 was monitored by real-time PCR during the time course of *in vitro L. major* infection in MDM from three new donors. Results indicate that miR-210 was crescendo up-regulated since 6 h post-infection in parasite infected macrophages (Fig. 4, panel A). This up-regulation was statistically significant at 12 and 24 h.

MiR-210 expression has been described to be mainly transcriptionally controlled by hypoxia-induced factor-1 alpha (HIF-1α) (reviewed in [48]). This TF was shown by us (Tiffin and Sysco Consortium, submitted paper) and by others [44], [49] to be activated in macrophages infected with *Leishmania*, probably through a hypoxia-independent pathway [44], [49]. In order to check if the observed up-regulation of miR-210 was specifically under HIF-1α control, we used siRNA to silence this TF before infection (HIF-1α silencing leads to a 90% decrease in HIF-1α protein abundance; Tiffin and Sysco Consortium, submitted paper). Levels of miR-210 were then monitored in macrophages incubated for 24 h with *L. major* parasites. In cells transfected with control siRNA, and as expected, miRNA-210 was overexpressed in infected cells in comparison to non-infected ones. (*p* = 0.001). HIF-1α silencing leads to a reduction by about 40% of miR-210 expression in non-infected cells (*p* = 0.001) and up to 60% in infected cells (*p* = 0.00002), indicating that induction of miR-210 is largely dependent on parasite-induced HIF-1α activation (Fig. 4, panel B). Noteworthy, the parasite load in HIF-1α silenced cells was drastically reduced, when compared to controls (Tiffin and Sysco Consortium, submitted paper). This is unlikely to be the result of any potential side effect activation by transfection agents, since the expression levels of several macrophage activation markers (TNF-α, IL-6, IL-8, iNOS and NO) were not significantly different between non transfected and transfected cells (data not shown).

Caspase-3 is considered as a key actor of apoptosis triggered by both the intrinsic and extrinsic pathways [46]. It is however well known that *Leishmania* spp. inhibit macrophage apoptosis [14] to ensure their intracellular differentiation and survival. To investigate if miR-210 up-regulation in infected macrophages might contribute to the anti-apoptotic behavior of *Leishmania*-infected macrophages through caspase-3 inhibition, we first measured pro-caspase 3 abundance in infected cells compared to non infected cells and then monitored the potential effect of miR-210 silencing on expression of this pro-apoptotic molecule. Figure 4, panel C shows a decreased abundance of pro-caspase-3 in macrophages infected with the parasite at 6, 12 and 24 h, strongly supporting the anti-apoptotic effect of *L. major* infection. However, miR-210 silencing did not affect the abundance of procaspase-3 (data not shown). This absence of effect could be attributed either to the partial effect of miR-210 silencing on its expression (46%, data not shown) although statistically significant (*p* = 0.0014), or to specific regulation of caspase-3 in MDM that differs from that observed in HeLa cells [46].

## Discussion

How host cells react to infection and how pathogens adapt to host cell microenvironment remain largely unsolved issues, though they are crucial for better understanding of host-pathogen interactions in order to set up efficient therapies. *Leishmania* parasites are among those pathogens that intriguingly contradict immune response dogma. When injected into skin, *Leishmania* promastigotes first interact with resident cells, i.e. PMN, dermal macrophages, keratinocytes, and Langerhans cells. Promastigotes are then rapidly phagocytized by PMN and macrophages and metamorphose to amastigotes. Ideally, ingested promastigotes are internalized in phagosomes that fuse with lysosomes, digest invaders and present their antigens to T cells to initiate adaptive responses. However, *Leishmania* parasites have evolved several escape mechanisms to subvert the innate immune responses and prevent the development of an efficient anti-parasitic response. Despite several studies that have been conducted on *Leishmania*-macrophage interaction, one should acknowledge that our understanding of the mechanisms deployed by *Leishmania* parasites to modulate the host cell's response to infection is still incomplete.

Several pathogens have been shown to modify cellular responses of their host through miRNAs, molecules now recognized as master regulators of major cellular processes. We therefore hypothesized that *L. major* parasites could alter the miRNA profile of infected macrophages during the first 24 h of their encounter.

Our study shows that about 20% of the tested miRNAs were specifically deregulated at different time points upon infection course, while their expression was unchanged in cells cultured in presence of inert latex beads. Several molecules that were differentially up-regulated at 3 and 6 h post-*L. major* infection, i.e., miR9, miR132, miR-146a, miR-155 and miR-187, are well known to control TLR-receptor signaling in monocytes [27], [50], [51]. Indeed, miR146a over-expression in human macrophages was reported to be noticeable as soon as 2 h after LPS treatment (acting through TLR4) [52]. A TLR5 agonist induced in these cells the same effect on miR-146a; in contrast agonists to TLR3, TLR7, and TLR9 had no effect.. In turn, miR-146a targets transcripts of TNF receptor-associated family (TRAF) 6 and IL-1 receptor-associated kinase (IRAK) 1, suggesting that miR-146a is a negative regulator of the fine-tuned inflammatory responses.

Induction of miR-155 is also a robust feature of the mammalian inflammatory responses in human [52] and murine [53] macrophage cell lines in response to LPS and other TLR ligands [54]. Interestingly, the two parallel TLR4-dependant cascades (Myd88 and TRIF) both contain miR-155-targets. Assisted by miR-155-regulated TAB2 [55], the MyD88 branch induces nuclear NF-κB translocation and AP-1 activation through IKK and MAPK, respectively. Although these observations suggest that miR-146 and miR-155 are most likely co- regulated, several studies have indicated that they might control the inflammatory response at different levels [56]. Inflammatory cytokines and/or TLR-responsive miRNA also include miR-132 [57] and miR-9; the latter directly targets the NF-κB1 providing a rapid and efficient negative feedback loop on NF-κB dependent pathways [50]. Although the entry mechanism of *Leishmania* is not fully elucidated, the up-regulation of several TLRs has been reported upon macrophage infection by parasites [58] with contradictory evidences in humans depending on the infecting *Leishmania* spp. Indeed, it was shown that *L.* (*Viannia*) *panamensis* infection results in up-regulation of TLR1, TLR2, TLR3, and TLR4 expression [59], inducing activation of infected macrophages, whereas infection with *L. donovani* suppresses the TLR2–NF-κB–mediated pro-inflammatory cytokine response [60]. In our hands, profiling of up-regulated and TLR-related miRNAs indicates that *L. major* infection preferentially induces activation of several TLR-dependent pathways (IRAK, TRAF-6, p50 NF-κB), in order to inhibit macrophage inflammatory responses. Altogether, several reports converge towards a model in which *Leishmania* parasites have developed different mechanisms to counteract the NF-κB-dependent inflammatory responses of infected macrophages (reviewed in [61]), as suggested by the absence of a broad range of cytokine and chemokine secretion accompanying the phagocytosis of *Leishmania* [62]. Among these mechanisms, one can cite i) the up-regulation of the A20 de-ubiquiting enzyme that counteracts the *L. donovani*-induced TRAF6 activation [60], ii) the *Leishmania* spp-induced specific cleavage of the p65 NF-κB subunit [63], [64] that we confirmed experimentally in *L. major*-infected human primary macrophages (data not shown), iii) the specific inhibition of p65 NF-κB subunit by *L. major* parasites [65], iv) the activation of the repressive p50 NF-κB homodimer by *L. amazonensis* [45], and v) the up-regulation of several miRNAs targeting the TLR-dependent pathway shown in this paper.

The experimentally validated identification of differentially expressed miRNAs targets highlighted several key molecules belonging to several pathways that play critical roles during the time course of infection. Besides TLR-2, TLR-4 and TLR-5 described above, TLR-related IL-1β, IL-6 and p50 NF-κB as well as pro-apoptotic targets have been identified as targets of up-regulated miRNA. This result is in keeping with our and others' early observations showing that transcripts of several apoptotic actors were down-modulated upon *L. major* infection of human macrophages [15].

Among other potential miRNA targets, we focused on selected chemokines transcripts. Indeed, although base pairing is not perfect in vertebrates, it is well known that miRNAs mainly act through degradation of their mRNA targets [66], [67], [68]. Based on the observation that five chemokines (CCL2, CCL5, CXCL10, CXCL11 and CXCL12) are targeted by *L. major*-regulated miRNAs i.e., Let-7a, miR-25, miR-26a, miR-132, miR-140, miR-146a and miR-155, we show a negative correlation of transcript abundance with their corresponding miRNAs. This result corroborates our early observation showing that expression levels of CCR2, CCL5 and CXCL10 mRNAs were drastically inhibited upon *L. major* infection of human macrophages [15].

It has been reported that tissue lesions of human cutaneous leishmaniasis due to *L. tropica* express high levels of intralesional iNOS and CCL2, indicating that NO likely promotes parasite killing by macrophages via CCL-2-mediated stimulation [69]. This result corroborates earlier observations showing that CCL2 acts synergistically with IFN-γ to antagonize IL-4 action, stimulate macrophage parasite-killing and promote healing [70]. Similarly, CCL2 enhances nitric oxide production and leishmanicidal activity of *L. infantum* infected macrophages [71]. These results suggest that inhibition of CCL2 (as a consequence of miRNA up-regulation reported in the present paper) might be a parasite-induced strategy to escape killing by macrophages. Our result showing inhibition of CXCL10 transcripts, however, contradicts earlier reports on the expression of high levels of this chemokine in CD14+ *L. braziliensis* infected-monocytes [72]. This discrepancy might be related to differences in *Leishmania* spp. or to cutaneous disease type [73].

This well-orchestrated mechanism is probably not the unique way for *L. major* parasites to escape killing by macrophages. Indeed, GO enrichment of deregulated miRNA potential targets showed several differences during time course infection in molecular functions, cellular components and biological processes.

Several miRNAs have been associated to regulation of apoptotic and anti-apoptotic pathways. Hence, miR-210 when inhibited increases the level of apoptosis in HeLa cells [46]; miR-22 promotes cell survival in UV irradiated cells through a tumor suppressor gene down-regulation [74]; down-regulation of miR-25 in ovarian cancer cells induces apoptosis [75]; miR-155 was described as having anti-apoptotic effects in murine macrophages during *Helicobacter pylori* infection [76]; and miR-133b is known to inhibit pro-survival molecules MCL-1 and Bcl-w proteins, two members of the BCL-2 family [47].

Being particularly interested in putative regulators of apoptotic and anti-apoptotic pathways (Tiffin and Sysco Consortium, submitted paper), we identified one particular microRNA, miR-210, that could possibly affect the abundance of apoptotic proteins like procaspase-3, a key actor of apoptosis triggered by both intrinsic and extrinsic pathways.

MiR-210 levels progressively and robustly increase through the time course of infection. Silencing experiments showed that its transcription is largely controlled by HIF-1α, a transcription factor directly related to hypoxia.

Although HIF-1α stabilization did not play any role in *L. donovani* entry in macrophages, its overexpression is beneficial to the parasite survival at the post-infective stage [44], [49], and its silencing reduces parasite load (Tiffin and Sysco Consortium, submitted paper). Interestingly, HIF-1α stabilization was not due to depletion of cellular oxygen levels and was unlikely a hypoxia-dependent phenomenon [44], [49]. It is however interesting to note that several miRNAs in addition to miRNA-210 i.e., miR-23, miR-24, miR-26a, miR-26b, miR-29a and miR-107 up-regulated through time course infection in our study were described as hypoxia-related [77], [78], negatively regulating HIF-1α through factor inhibiting-HIF-1α (FIH) [79] or induced by this TF [80].

To our knowledge, our study is the first one showing miR-210 induction in response to a pathogen; further investigation is warranted in order to clarify the biological significance of this up-regulation in response to *L. major* infection.

It has been well known for almost two decades that *Leishmania* infection inhibits macrophage apoptosis [14]. The parasite-induced anti-apoptotic effect is associated with a significant reduction of caspase-3 activity in *L. major*- or *L. mexicana*-infected PMN [81] or monocyte-derived dendritic cells [82]. In addition, *L. infantum* infection affected the apoptosis of U-937 human monocytic cell line via a mechanism involving inhibition of caspase-3 activation [83]. Conversely, it was also shown that silencing of miR-210 in HeLa cells induced caspase-3 activity [46]. We hence hypothesized that pro-caspase3 could be targeted by miR-210 in infected human MDM, thereby inhibiting their apoptosis. Although silencing miR-210 in infected macrophages did not reverse the *L. major-*induced pro-caspase-3 inhibition, we cannot exclude that this anti-apoptotic function of miR-210 takes place through the targeting of other pro-apoptotic molecules. Indeed, miR-210 has been shown to promote cell survival by targeting caspase-8-associated protein 2 in rat mesenchymal stem cells [84], E2F3 transcription factor in human pulmonary artery smooth muscle cell [85] and apoptosis-inducing factor, mitochondrion-associated, 3 (AIFM3) in hypoxic human hepatoma cells [86]. However, the biological significance of *L. major*-induced miR-210 may lie in non-apoptotic biological processes, as miR-210 has been recently described to down-regulate NF-κB1 (the p105 precursor of p50 NF-κB subunit) [87].

When reporting our results, a recent study has elegantly shown that *L. donovani* infection down-regulates expression of miR-122 and genes involved in cholesterol biosynthesis in infected mouse livers. This deregulation was conducted through *Leishmania* metalloprotease gp63, which inhibits Dicer1-mediated pre-miR-122 processing upon Dicer1 degradation in infected cells [88]. *Leishmania* virulence factors or other parasit*e* exosome components involved in cell-cell contact might also be involved in mir-210 up-regulation in human macrophage upon *L. major* infection.

### Conclusion

In conclusion, we report for the first time that within the first 24 h of infection by *L. major* the miRNA profile of human primary macrophages is strongly and rapidly modified.

Alterations in miRNA levels likely reflect the remarkable capacity of parasites to modify the host responses to ensure their intracellular differentiation and multiplication.

## Supporting Information

Figure S1 **Principal component analysis of miRNA expression profiles raised with the whole 365 miRNAs set (upper panel) or with only deregulated sets (lower panel).** These figures show similarities of miRNA profiles between the three donors at different time points upon infection (panels A and E: 3 h post infection; panels B and F: 6 h post infection; panels C and G: 12 h post infection and panels D and H: 24 h post infection).
(TIF)

Figure S2 **Hierarchical cluster analyses of deregulated miRNA expression in** ***L. major*** **-infected human primary macrophages at different time points upon infection.** The miRNA expression values are presented using a red-white-blue color scheme, with red data points indicating higher expression than median values, white indicating expression equal to the median, and blue indicating lower expression than the median. MiRNAs were analyzed independently based on their expression before and upon infection at different time points (3, 6, 12 and 24 h) of primary human macrophages from three healthy donors (D1, D2 and D3).
(TIF)

Figure S3 **Individual PCR validation of a selected set of deregulated miRNAs in** ***L. major*** **-infected human macrophages.** Scatter plot analysis shows correlation between mean expression levels of nine miRNAs measured by array analysis (PCR array) and mean expression levels tested using individual qRT-PCR (Individual PCR) in three donors. Correlation coefficient *r* and statistical *p* values are indicated. Results were expressed using the 2^−ΔΔCt^ method.
(TIFF)

Figure S4 **Molecular functions and cellular components of** ***L. major*** **-infected human primary macrophage miRNA-targets at 3 h post-infection.** Regulatory network was obtained after GO enrichment deduced from analysis of up- or down-regulated miRNA-targets. Yellow color gradient intensity correlates with up- or down-regulation levels. White nodes are not significantly overrepresented. The area of each node is proportional to the number of genes in the set annotated to the corresponding GO category. Interactions were visualized as a network using Cytoscape and BINGO plugin.
(TIF)

Figure S5 **Molecular functions and cellular components of** ***L. major*** **-infected human primary macrophage miRNA-targets at 6 h post-infection.** Regulatory network was obtained after GO enrichment deduced from analysis of up- or down-regulated miRNA-targets. Yellow color gradient intensity correlates with up- or down-regulation levels. White nodes are not significantly overrepresented. The area of each node is proportional to the number of genes in the set annotated to the corresponding GO category. Interactions were visualized as a network using Cytoscape and BINGO plugin.
(TIF)

Figure S6 **Molecular functions and cellular components of** ***L. major*** **-infected human primary macrophage miRNA-targets at 12 h post-infection.** Regulatory network was obtained after GO enrichment deduced from analysis of up- or down-regulated miRNA-targets. Yellow color gradient intensity correlates with up- or down-regulation levels. White nodes are not significantly overrepresented. The area of each node is proportional to the number of genes in the set annotated to the corresponding GO category. Interactions were visualized as a network using Cytoscape and BINGO plugin.
(TIF)

Figure S7 **Molecular functions and cellular components of** ***L. major*** **-infected human primary macrophage miRNA-targets at 24 h post-infection.** Regulatory network was obtained after GO enrichment deduced from analysis of up- or down-regulated miRNA-targets. Yellow color gradient intensity correlates with up- or down-regulation levels. White nodes are not significantly overrepresented. The area of each node is proportional to the number of genes in the set annotated to the corresponding GO category. Interactions were visualized as a network using Cytoscape and BINGO plugin.
(TIF)

Figure S8 **Biological processes deduced from analysis of deregulated miRNA-targets in** ***L. major*** **-infected human macrophages at 6 h post-infection.** Yellow color gradient intensity correlates with up- or down-regulation levels. White nodes are not significantly overrepresented. The area of each node is proportional to the number of genes in the set annotated to the corresponding GO category. Interactions were visualized as a network using Cytoscape and BINGO plugin.
(TIF)

Figure S9 **Biological processes deduced from analysis of deregulated miRNA-targets in** ***L. major*** **-infected human macrophages at 12 h post-infection.** Yellow color gradient intensity correlates with up- or down-regulation levels. White nodes are not significantly overrepresented. The area of each node is proportional to the number of genes in the set annotated to the corresponding GO category. Interactions were visualized as a network using Cytoscape and BINGO plugin.
(TIF)

Figure S10 **Biological processes deduced from analysis of deregulated miRNA-targets in** ***L. major*** **-infected human macrophages at 24 h post-infection.** Yellow color gradient intensity correlates with up- or down-regulation levels. White nodes are not significantly overrepresented. The area of each node is proportional to the number of genes in the set annotated to the corresponding GO category. Interactions were visualized as a network using Cytoscape and BINGO plugin.
(TIF)

Figure S11 **Scatter plot analysis showing a negative correlation between expressions of an up-regulated set of miRNAs and their targeted chemokine transcripts.** Expression of let-7a, miR-25, miR-26a, miR-140, miR-146a and miR-155 at 3 h and miR-23b and miR-132 at 6 h post-infection of three healthy donors (D1, D2 and D3) is negatively correlated with CCL2, CCL5, CXCL10, CXCL11 and CXCL12 mRNA levels at 12 and 24 h post-infection in *L. major*-infected human macrophages. Correlation coefficient *r* and statistical *p* values are indicated. Results were expressed using the 2^−ΔΔCt^ method.
(TIFF)

Table S1 **Expression levels of 365 human miRNAs in** ***L. major*** **-infected and latex beads-incubated human primary macrophages.** Results are obtained at different time points (3, 6, 12 and 24 h) in macrophages from three healthy donors (D1, D2 and D3). Analysis was assessed by qRT-PCR and results were expressed using the 2^−ΔΔCt^ method. When signals are either undetectable or below the background, they are indicated as (ND).
(XLS)

Table S2 **Identification of differentially regulated-miRNA targets.** Different lists were generated using miRWalk database using miRNAs identified as differentially up- or down-regulated at 3, 6, 12 and 24 h post-infection in *L. major*-infected primary human macrophages. Tables indicate the miRNA name, gene name, entrez ID and Pubmed ID experimental validation of their targets.
(XLS)

Table S3 **Identification of differentially regulated-miRNAs up-stream regulating transcription factors.** Different lists were generated using TransmiR database using miRNAs identified as differentially up- or down-regulated at 3, 6, 12 and 24 h post-infection in *L. major*-infected primary human macrophages. Tables indicate the transcription factor/signaling component, its entrez ID and type of activity on miRNA, miRNA name and Pubmed ID experimental validation of indicated up-stream regulation.
(XLS)

## References

[1] Murray HW, Berman JD, Davies CR, Saravia NG (2005) Advances in leishmaniasis. Lancet 366: 1561–1577. 16257344 10.1016/S0140-6736(05)67629-5

[2] Duclos S, Desjardins M (2000) Subversion of a young phagosome: the survival strategies of intracellular pathogens. Cell Microbiol 2: 365–377. 11207592 10.1046/j.1462-5822.2000.00066.x

[3] Olivier M, Gregory DJ, Forget G (2005) Subversion mechanisms by which Leishmania parasites can escape the host immune response: a signaling point of view. Clin Microbiol Rev 18: 293–305. 15831826 10.1128/CMR.18.2.293-305.2005 PMC1082797

[4] Peters NC, Sacks DL (2009) The impact of vector-mediated neutrophil recruitment on cutaneous leishmaniasis. Cell Microbiol 11: 1290–1296. 19545276 10.1111/j.1462-5822.2009.01348.x PMC3431610

[5] Moreno I, Dominguez M, Cabanes D, Aizpurua C, Torano A (2010) Kinetic analysis of ex vivo human blood infection by Leishmania. PLoS Negl Trop Dis 4: e743. 20644618 10.1371/journal.pntd.0000743 PMC2903471

[6] Reiner NE, Ng W, McMaster WR (1987) Parasite-accessory cell interactions in murine leishmaniasis. II. Leishmania donovani suppresses macrophage expression of class I and class II major histocompatibility complex gene products. J Immunol 138: 1926–1932. 2434567

[7] Kaye PM, Rogers NJ, Curry AJ, Scott JC (1994) Deficient expression of co-stimulatory molecules on Leishmania-infected macrophages. Eur J Immunol 24: 2850–2854. 7525308 10.1002/eji.1830241140

[8] Bhardwaj S, Srivastava N, Sudan R, Saha B (2010) Leishmania interferes with host cell signaling to devise a survival strategy. J Biomed Biotechnol 2010: 109189. 20396387 10.1155/2010/109189 PMC2852600

[9] Shadab M, Ali N (2011) Evasion of Host Defence by Leishmania donovani: Subversion of Signaling Pathways. Mol Biol Int 2011: 343961. 22091401 10.4061/2011/343961 PMC3199940

[10] Shio MT, Hassani K, Isnard A, Ralph B, Contreras I, et al (2012) Host cell signalling and leishmania mechanisms of evasion. J Trop Med 2012: 819512. 22131998 10.1155/2012/819512 PMC3216306

[11] Bogdan C (2008) Mechanisms and consequences of persistence of intracellular pathogens: leishmaniasis as an example. Cell Microbiol 10: 1221–1234. 18363880 10.1111/j.1462-5822.2008.01146.x

[12] Teixeira MJ, Teixeira CR, Andrade BB, Barral-Netto M, Barral A (2006) Chemokines in host-parasite interactions in leishmaniasis. Trends Parasitol 22: 32–40. 16310413 10.1016/j.pt.2005.11.010

[13] McConville MJ, Naderer T (2011) Metabolic pathways required for the intracellular survival of Leishmania. Annu Rev Microbiol 65: 543–561. 21721937 10.1146/annurev-micro-090110-102913

[14] Moore KJ, Matlashewski G (1994) Intracellular infection by Leishmania donovani inhibits macrophage apoptosis. J Immunol 152: 2930–2937. 8144893

[15] Guerfali FZ, Laouini D, Guizani-Tabbane L, Ottones F, Ben-Aissa K, et al (2008) Simultaneous gene expression profiling in human macrophages infected with Leishmania major parasites using SAGE. BMC Genomics 9: 238. 18495030 10.1186/1471-2164-9-238 PMC2430024

[16] Akarid K, Arnoult D, Micic-Polianski J, Sif J, Estaquier J, et al (2004) Leishmania major-mediated prevention of programmed cell death induction in infected macrophages is associated with the repression of mitochondrial release of cytochrome c. J Leukoc Biol 76: 95–103. 15075349 10.1189/jlb.1001877

[17] Ruhland A, Leal N, Kima PE (2007) Leishmania promastigotes activate PI3K/Akt signalling to confer host cell resistance to apoptosis. Cell Microbiol 9: 84–96. 16889626 10.1111/j.1462-5822.2006.00769.x

[18] Barbato C, Arisi I, Frizzo ME, Brandi R, Da Sacco L, et al (2009) Computational challenges in miRNA target predictions: to be or not to be a true target? J Biomed Biotechnol 2009: 803069. 19551154 10.1155/2009/803069 PMC2699446

[19] Krek A, Grun D, Poy MN, Wolf R, Rosenberg L, et al (2005) Combinatorial microRNA target predictions. Nat Genet 37: 495–500. 15806104 10.1038/ng1536

[20] Lewis BP, Burge CB, Bartel DP (2005) Conserved seed pairing, often flanked by adenosines, indicates that thousands of human genes are microRNA targets. Cell 120: 15–20. 15652477 10.1016/j.cell.2004.12.035

[21] Scaria V, Hariharan M, Maiti S, Pillai B, Brahmachari SK (2006) Host-virus interaction: a new role for microRNAs. Retrovirology 3: 68. 17032463 10.1186/1742-4690-3-68 PMC1626483

[22] Skalsky RL, Cullen BR (2010) Viruses, microRNAs, and host interactions. Annu Rev Microbiol 64: 123–141. 20477536 10.1146/annurev.micro.112408.134243 PMC3621958

[23] Katiyar-Agarwal S, Jin H (2010) Role of small RNAs in host-microbe interactions. Annu Rev Phytopathol 48: 225–246. 20687832 10.1146/annurev-phyto-073009-114457 PMC3752435

[24] Eulalio A, Schulte L, Vogel J (2012) The mammalian microRNA response to bacterial infections. RNA Biol 9: 742–750. 22664920 10.4161/rna.20018

[25] Hakimi MA, Cannella D (2011) Apicomplexan parasites and subversion of the host cell microRNA pathway. Trends Parasitol 27: 481–486. 21840260 10.1016/j.pt.2011.07.001

[26] Zeiner GM, Norman KL, Thomson JM, Hammond SM, Boothroyd JC (2010) Toxoplasma gondii infection specifically increases the levels of key host microRNAs. PLoS One 5: e8742. 20090903 10.1371/journal.pone.0008742 PMC2806928

[27] Nahid MA, Satoh M, Chan EK (2011) MicroRNA in TLR signaling and endotoxin tolerance. Cell Mol Immunol 8: 388–403. 21822296 10.1038/cmi.2011.26 PMC3618661

[28] Liu Y, Kao WJ (2002) Human macrophage adhesion on fibronectin: the role of substratum and intracellular signalling kinases. Cell Signal 14: 145–152. 11781139 10.1016/s0898-6568(01)00246-7

[29] Spath GF, Beverley SM (2001) A lipophosphoglycan-independent method for isolation of infective Leishmania metacyclic promastigotes by density gradient centrifugation. Exp Parasitol 99: 97–103. 11748963 10.1006/expr.2001.4656

[30] Hulten LM, Olson FJ, Aberg H, Carlsson J, Karlstrom L, et al (2010) 15-Lipoxygenase-2 is expressed in macrophages in human carotid plaques and regulated by hypoxia-inducible factor-1alpha. Eur J Clin Invest 40: 11–17. 19912316 10.1111/j.1365-2362.2009.02223.x

[31] Daigneault M, Preston JA, Marriott HM, Whyte MK, Dockrell DH (2010) The identification of markers of macrophage differentiation in PMA-stimulated THP-1 cells and monocyte-derived macrophages. PLoS One 5: e8668. 20084270 10.1371/journal.pone.0008668 PMC2800192

[32] Vandesompele J, De Preter K, Pattyn F, Poppe B, Van Roy N, et al (2002) Accurate normalization of real-time quantitative RT-PCR data by geometric averaging of multiple internal control genes. Genome Biol 3: RESEARCH0034. 12184808 10.1186/gb-2002-3-7-research0034 PMC126239

[33] Saeed AI, Sharov V, White J, Li J, Liang W, et al (2003) TM4: a free, open-source system for microarray data management and analysis. Biotechniques 34: 374–378. 12613259 10.2144/03342mt01

[34] Dweep H, Sticht C, Pandey P, Gretz N (2011) miRWalk–database: prediction of possible miRNA binding sites by “walking” the genes of three genomes. J Biomed Inform 44: 839–847. 21605702 10.1016/j.jbi.2011.05.002

[35] Lynn DJ, Winsor GL, Chan C, Richard N, Laird MR, et al (2008) InnateDB: facilitating systems-level analyses of the mammalian innate immune response. Mol Syst Biol 4: 218. 18766178 10.1038/msb.2008.55 PMC2564732

[36] Maere S, Heymans K, Kuiper M (2005) BiNGO: a Cytoscape plugin to assess overrepresentation of gene ontology categories in biological networks. Bioinformatics 21: 3448–3449. 15972284 10.1093/bioinformatics/bti551

[37] Shannon P, Markiel A, Ozier O, Baliga NS, Wang JT, et al (2003) Cytoscape: a software environment for integrated models of biomolecular interaction networks. Genome Res 13: 2498–2504. 14597658 10.1101/gr.1239303 PMC403769

[38] Smoot ME, Ono K, Ruscheinski J, Wang PL, Ideker T (2011) Cytoscape 2.8: new features for data integration and network visualization. Bioinformatics 27: 431–432. 21149340 10.1093/bioinformatics/btq675 PMC3031041

[39] Wang J, Lu M, Qiu C, Cui Q (2010) TransmiR: a transcription factor-microRNA regulation database. Nucleic Acids Res 38: D119–122. 19786497 10.1093/nar/gkp803 PMC2808874

[40] Whitney AR, Diehn M, Popper SJ, Alizadeh AA, Boldrick JC, et al (2003) Individuality and variation in gene expression patterns in human blood. Proc Natl Acad Sci U S A 100: 1896–1901. 12578971 10.1073/pnas.252784499 PMC149930

[41] Turan N, Katari S, Coutifaris C, Sapienza C (2010) Explaining inter-individual variability in phenotype: is epigenetics up to the challenge? Epigenetics 5: 16–19. 20083905 10.4161/epi.5.1.10557 PMC2829373

[42] Stratz C, Nuhrenberg TG, Binder H, Valina CM, Trenk D, et al (2012) Micro-array profiling exhibits remarkable intra-individual stability of human platelet micro-RNA. Thromb Haemost 107: 634–641. 22371016 10.1160/TH11-10-0742

[43] Nandan D, Camargo de Oliveira C, Moeenrezakhanlou A, Lopez M, Silverman JM, et al (2012) Myeloid cell IL-10 production in response to leishmania involves inactivation of glycogen synthase kinase-3beta downstream of phosphatidylinositol-3 kinase. J Immunol 188: 367–378. 22140263 10.4049/jimmunol.1100076

[44] Singh AK, Mukhopadhyay C, Biswas S, Singh VK, Mukhopadhyay CK (2012) Intracellular pathogen Leishmania donovani activates hypoxia inducible factor-1 by dual mechanism for survival advantage within macrophage. PLoS One 7: e38489. 22701652 10.1371/journal.pone.0038489 PMC3373497

[45] Calegari-Silva TC, Pereira RM, De-Melo LD, Saraiva EM, Soares DC, et al (2009) NF-kappaB-mediated repression of iNOS expression in Leishmania amazonensis macrophage infection. Immunol Lett 127: 19–26. 19712696 10.1016/j.imlet.2009.08.009

[46] Cheng AM, Byrom MW, Shelton J, Ford LP (2005) Antisense inhibition of human miRNAs and indications for an involvement of miRNA in cell growth and apoptosis. Nucleic Acids Res 33: 1290–1297. 15741182 10.1093/nar/gki200 PMC552951

[47] Crawford M, Batte K, Yu L, Wu X, Nuovo GJ, et al (2009) MicroRNA 133B targets pro-survival molecules MCL-1 and BCL2L2 in lung cancer. Biochem Biophys Res Commun 388: 483–489. 19654003 10.1016/j.bbrc.2009.07.143 PMC2824514

[48] Chan YC, Banerjee J, Choi SY, Sen CK (2012) miR-210: the master hypoxamir. Microcirculation 19: 215–223. 22171547 10.1111/j.1549-8719.2011.00154.x PMC3399423

[49] Degrossoli A, Bosetto MC, Lima CB, Giorgio S (2007) Expression of hypoxia-inducible factor 1alpha in mononuclear phagocytes infected with Leishmania amazonensis. Immunol Lett 114: 119–125. 17983667 10.1016/j.imlet.2007.09.009

[50] Bazzoni F, Rossato M, Fabbri M, Gaudiosi D, Mirolo M, et al (2009) Induction and regulatory function of miR-9 in human monocytes and neutrophils exposed to proinflammatory signals. Proc Natl Acad Sci U S A 106: 5282–5287. 19289835 10.1073/pnas.0810909106 PMC2664036

[51] Quinn SR, O'Neill LA (2011) A trio of microRNAs that control Toll-like receptor signalling. Int Immunol 23: 421–425. 21652514 10.1093/intimm/dxr034

[52] Taganov KD, Boldin MP, Chang KJ, Baltimore D (2006) NF-kappaB-dependent induction of microRNA miR-146, an inhibitor targeted to signaling proteins of innate immune responses. Proc Natl Acad Sci U S A 103: 12481–12486. 16885212 10.1073/pnas.0605298103 PMC1567904

[53] Tili E, Michaille JJ, Cimino A, Costinean S, Dumitru CD, et al (2007) Modulation of miR-155 and miR-125b levels following lipopolysaccharide/TNF-alpha stimulation and their possible roles in regulating the response to endotoxin shock. J Immunol 179: 5082–5089. 17911593 10.4049/jimmunol.179.8.5082

[54] O'Connell RM, Taganov KD, Boldin MP, Cheng G, Baltimore D (2007) MicroRNA-155 is induced during the macrophage inflammatory response. Proc Natl Acad Sci U S A 104: 1604–1609. 17242365 10.1073/pnas.0610731104 PMC1780072

[55] Ceppi M, Pereira PM, Dunand-Sauthier I, Barras E, Reith W, et al (2009) MicroRNA-155 modulates the interleukin-1 signaling pathway in activated human monocyte-derived dendritic cells. Proc Natl Acad Sci U S A 106: 2735–2740. 19193853 10.1073/pnas.0811073106 PMC2650335

[56] Schulte LN, Westermann AJ, Vogel J (2012) Differential activation and functional specialization of miR-146 and miR-155 in innate immune sensing. Nucleic Acids Res 41: 542–553. 23143100 10.1093/nar/gks1030 PMC3592429

[57] Wanet A, Tacheny A, Arnould T, Renard P (2012) miR-212/132 expression and functions: within and beyond the neuronal compartment. Nucleic Acids Res 40: 4742–4753. 22362752 10.1093/nar/gks151 PMC3367188

[58] Faria MS, Reis FC, Lima AP (2012) Toll-like receptors in leishmania infections: guardians or promoters? J Parasitol Res 2012: 930257. 22523644 10.1155/2012/930257 PMC3317170

[59] Gallego C, Golenbock D, Gomez MA, Saravia NG (2011) Toll-like receptors participate in macrophage activation and intracellular control of Leishmania (Viannia) panamensis. Infect Immun 79: 2871–2879. 21518783 10.1128/IAI.01388-10 PMC3191987

[60] Srivastav S, Kar S, Chande AG, Mukhopadhyaya R, Das PK (2012) Leishmania donovani exploits host deubiquitinating enzyme A20, a negative regulator of TLR signaling, to subvert host immune response. J Immunol 189: 924–934. 22685311 10.4049/jimmunol.1102845

[61] Reinhard K, Huber M, Lohoff M, Visekruna A (2012) The role of NF-kappaB activation during protection against Leishmania infection. Int J Med Microbiol 302: 230–235. 22901377 10.1016/j.ijmm.2012.07.006

[62] Ji J, Sun J, Soong L (2003) Impaired expression of inflammatory cytokines and chemokines at early stages of infection with Leishmania amazonensis. Infect Immun 71: 4278–4288. 12874303 10.1128/IAI.71.8.4278-4288.2003 PMC166010

[63] Gregory DJ, Godbout M, Contreras I, Forget G, Olivier M (2008) A novel form of NF-kappaB is induced by Leishmania infection: involvement in macrophage gene expression. Eur J Immunol 38: 1071–1081. 18383035 10.1002/eji.200737586

[64] Neves BM, Silvestre R, Resende M, Ouaissi A, Cunha J, et al (2010) Activation of phosphatidylinositol 3-kinase/Akt and impairment of nuclear factor-kappaB: molecular mechanisms behind the arrested maturation/activation state of Leishmania infantum-infected dendritic cells. Am J Pathol 177: 2898–2911. 21037075 10.2353/ajpath.2010.100367 PMC2993270

[65] Guizani-Tabbane L, Ben-Aissa K, Belghith M, Sassi A, Dellagi K (2004) Leishmania major amastigotes induce p50/c-Rel NF-kappa B transcription factor in human macrophages: involvement in cytokine synthesis. Infect Immun 72: 2582–2589. 15102766 10.1128/IAI.72.5.2582-2589.2004 PMC387864

[66] Filipowicz W, Bhattacharyya SN, Sonenberg N (2008) Mechanisms of post-transcriptional regulation by microRNAs: are the answers in sight? Nat Rev Genet 9: 102–114. 18197166 10.1038/nrg2290

[67] Guo H, Ingolia NT, Weissman JS, Bartel DP (2010) Mammalian microRNAs predominantly act to decrease target mRNA levels. Nature 466: 835–840. 20703300 10.1038/nature09267 PMC2990499

[68] Huntzinger E, Izaurralde E (2011) Gene silencing by microRNAs: contributions of translational repression and mRNA decay. Nat Rev Genet 12: 99–110. 21245828 10.1038/nrg2936

[69] Kumar R, Bumb RA, Salotra P (2010) Evaluation of localized and systemic immune responses in cutaneous leishmaniasis caused by Leishmania tropica: interleukin-8, monocyte chemotactic protein-1 and nitric oxide are major regulatory factors. Immunology 130: 193–201. 20102417 10.1111/j.1365-2567.2009.03223.x PMC2878464

[70] Ritter U, Moll H (2000) Monocyte chemotactic protein-1 stimulates the killing of leishmania major by human monocytes, acts synergistically with IFN-gamma and is antagonized by IL-4. Eur J Immunol 30: 3111–3120. 11093125 10.1002/1521-4141(200011)30:11<3111::AID-IMMU3111>3.0.CO;2-O

[71] Brandonisio O, Panaro MA, Fumarola I, Sisto M, Leogrande D, et al (2002) Macrophage chemotactic protein-1 and macrophage inflammatory protein-1 alpha induce nitric oxide release and enhance parasite killing in Leishmania infantum-infected human macrophages. Clin Exp Med 2: 125–129. 12447609 10.1007/s102380200017

[72] Vargas-Inchaustegui DA, Hogg AE, Tulliano G, Llanos-Cuentas A, Arevalo J, et al (2010) CXCL10 production by human monocytes in response to Leishmania braziliensis infection. Infect Immun 78: 301–308. 19901067 10.1128/IAI.00959-09 PMC2798186

[73] Ritter U, Korner H (2002) Divergent expression of inflammatory dermal chemokines in cutaneous leishmaniasis. Parasite Immunol 24: 295–301. 12102714 10.1046/j.1365-3024.2002.00467.x

[74] Tan G, Shi Y, Wu ZH (2012) MicroRNA-22 promotes cell survival upon UV radiation by repressing PTEN. Biochem Biophys Res Commun 417: 546–551. 22166214 10.1016/j.bbrc.2011.11.160 PMC3259290

[75] Zhang H, Zuo Z, Lu X, Wang L, Wang H, et al (2012) MiR-25 regulates apoptosis by targeting Bim in human ovarian cancer. Oncol Rep 27: 594–598. 22076535 10.3892/or.2011.1530

[76] Koch M, Mollenkopf HJ, Klemm U, Meyer TF (2012) Induction of microRNA-155 is TLR- and type IV secretion system-dependent in macrophages and inhibits DNA-damage induced apoptosis. Proc Natl Acad Sci U S A 109: E1153–1162. 22509021 10.1073/pnas.1116125109 PMC3358876

[77] Kulshreshtha R, Ferracin M, Wojcik SE, Garzon R, Alder H, et al (2007) A microRNA signature of hypoxia. Mol Cell Biol 27: 1859–1867. 17194750 10.1128/MCB.01395-06 PMC1820461

[78] Yang Z, Wu L, Zhu X, Xu J, Jin R, et al (2013) MiR-29a modulates the angiogenic properties of human endothelial cells. Biochem Biophys Res Commun 434: 143–149. 23541945 10.1016/j.bbrc.2013.03.054 PMC3646542

[79] Peng H, Hamanaka RB, Katsnelson J, Hao LL, Yang W, et al (2012) MicroRNA-31 targets FIH-1 to positively regulate corneal epithelial glycogen metabolism. FASEB J 26: 3140–3147. 22532441 10.1096/fj.11-198515 PMC3405266

[80] Babar IA, Czochor J, Steinmetz A, Weidhaas JB, Glazer PM, et al (2011) Inhibition of hypoxia-induced miR-155 radiosensitizes hypoxic lung cancer cells. Cancer Biol Ther 12: 908–914. 22027557 10.4161/cbt.12.10.17681 PMC3280906

[81] Aga E, Katschinski DM, van Zandbergen G, Laufs H, Hansen B, et al (2002) Inhibition of the spontaneous apoptosis of neutrophil granulocytes by the intracellular parasite Leishmania major. J Immunol 169: 898–905. 12097394 10.4049/jimmunol.169.2.898

[82] Valdes-Reyes L, Argueta J, Moran J, Salaiza N, Hernandez J, et al (2009) Leishmania mexicana: inhibition of camptothecin-induced apoptosis of monocyte-derived dendritic cells. Exp Parasitol 121: 199–207. 19041644 10.1016/j.exppara.2008.10.020

[83] Lisi S, Sisto M, Acquafredda A, Spinelli R, Schiavone M, et al (2005) Infection with Leishmania infantum Inhibits actinomycin D-induced apoptosis of human monocytic cell line U-937. J Eukaryot Microbiol 52: 211–217. 15926996 10.1111/j.1550-7408.2005.00026.x

[84] Kim HW, Haider HK, Jiang S, Ashraf M (2009) Ischemic preconditioning augments survival of stem cells via miR-210 expression by targeting caspase-8-associated protein 2. J Biol Chem 284: 33161–33168. 19721136 10.1074/jbc.M109.020925 PMC2785158

[85] Gou D, Ramchandran R, Peng X, Yao L, Kang K, et al (2012) miR-210 has an anti-apoptotic effect in pulmonary artery smooth muscle cells during hypoxia. Am J Physiol Lung Cell Mol Physiol 10.1152/ajplung.00344.2011 PMC3469638 22886504

[86] Yang W, Sun T, Cao J, Liu F, Tian Y, et al (2012) Downregulation of miR-210 expression inhibits proliferation, induces apoptosis and enhances radiosensitivity in hypoxic human hepatoma cells in vitro. Exp Cell Res 318: 944–954. 22387901 10.1016/j.yexcr.2012.02.010

[87] Qi J, Qiao Y, Wang P, Li S, Zhao W, et al (2012) microRNA-210 negatively regulates LPS-induced production of proinflammatory cytokines by targeting NF-kappaB1 in murine macrophages. FEBS Lett 586: 1201–1207. 22575656 10.1016/j.febslet.2012.03.011

[88] Ghosh J, Bose M, Roy S, Bhattacharyya SN (2013) Leishmania donovani targets Dicer1 to downregulate miR-122, lower serum cholesterol, and facilitate murine liver infection. Cell Host Microbe 13: 277–288. 23498953 10.1016/j.chom.2013.02.005 PMC3605572

