# Supplementary figures and images for: MicroRNA Expression Profile in Human Macrophages in Response to Leishmania major Infection
Source: PLoS Negl Trop Dis. 2013 Oct 3;7(10):e2478. doi: 10.1371/journal.pntd.0002478 (PMC3789763; doi:10.1371/journal.pntd.0002478)

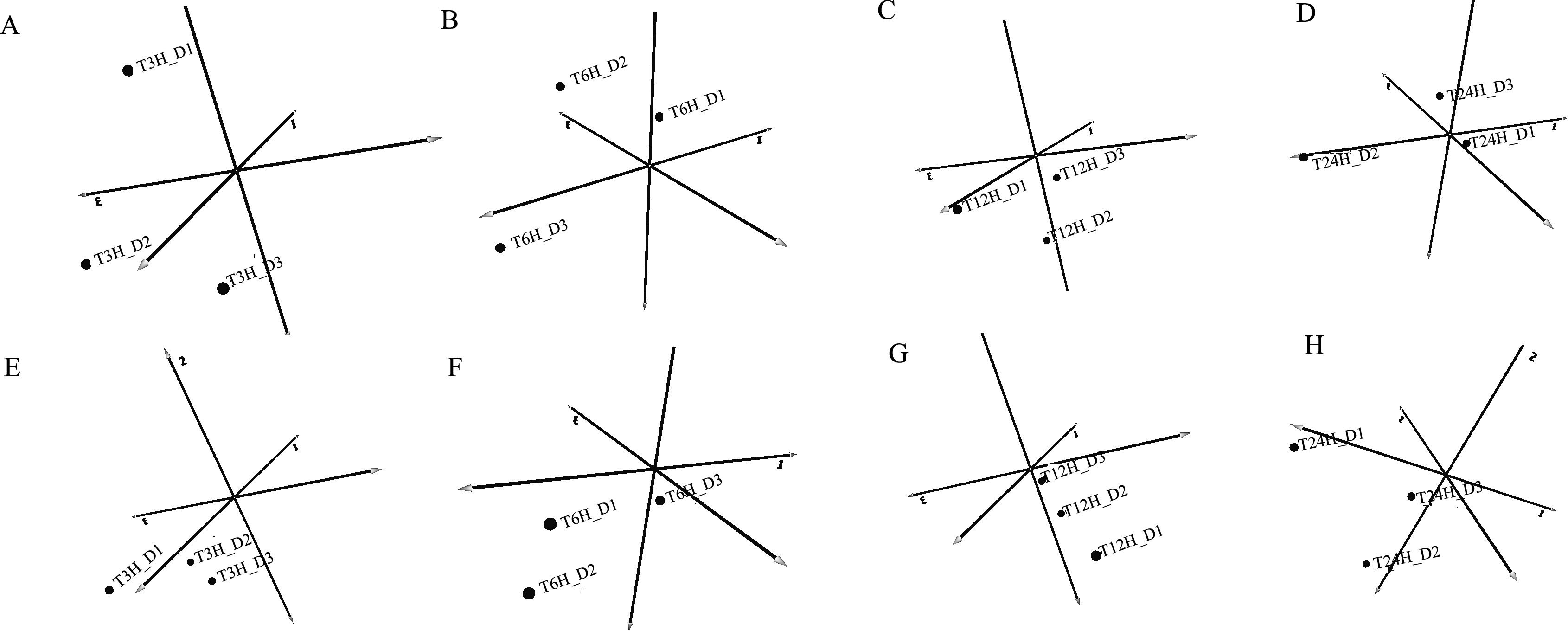

Supplement: Figure S1 — Principal component analysis of miRNA expression profiles raised with the whole 365 miRNAs set (upper panel) or with only deregulated sets (lower panel). These figures show similarities of miRNA profiles between the three donors at different time points upon infection (panels A and E: 3 h post infection; panels B and F: 6 h post infection; panels C and G: 12 h post infection and panels D and H: 24 h post infection). (TIF) [file pntd.0002478.s001.tif]

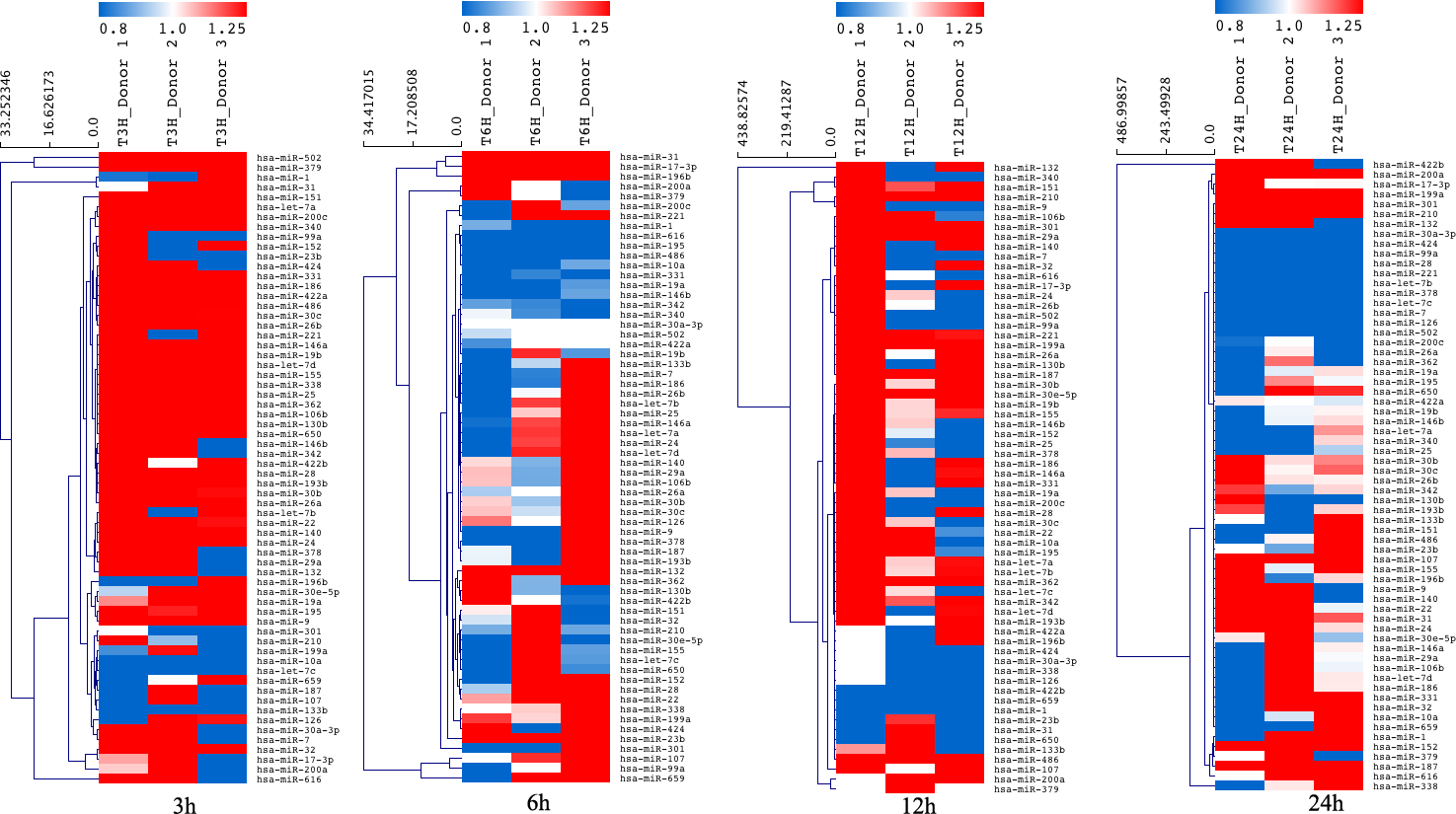

Supplement: Figure S2 — Hierarchical cluster analyses of deregulated miRNA expression in L. major -infected human primary macrophages at different time points upon infection. The miRNA expression values are presented using a red-white-blue color scheme, with red data points indicating higher expression than median values, white indicating expression equal to the median, and blue indicating lower expression than the median. MiRNAs were analyzed independently based on their expression before and upon infection at different time points (3, 6, 12 and 24 h) of primary human macrophages from three healthy donors (D1, D2 and D3). (TIF) [file pntd.0002478.s002.tif]

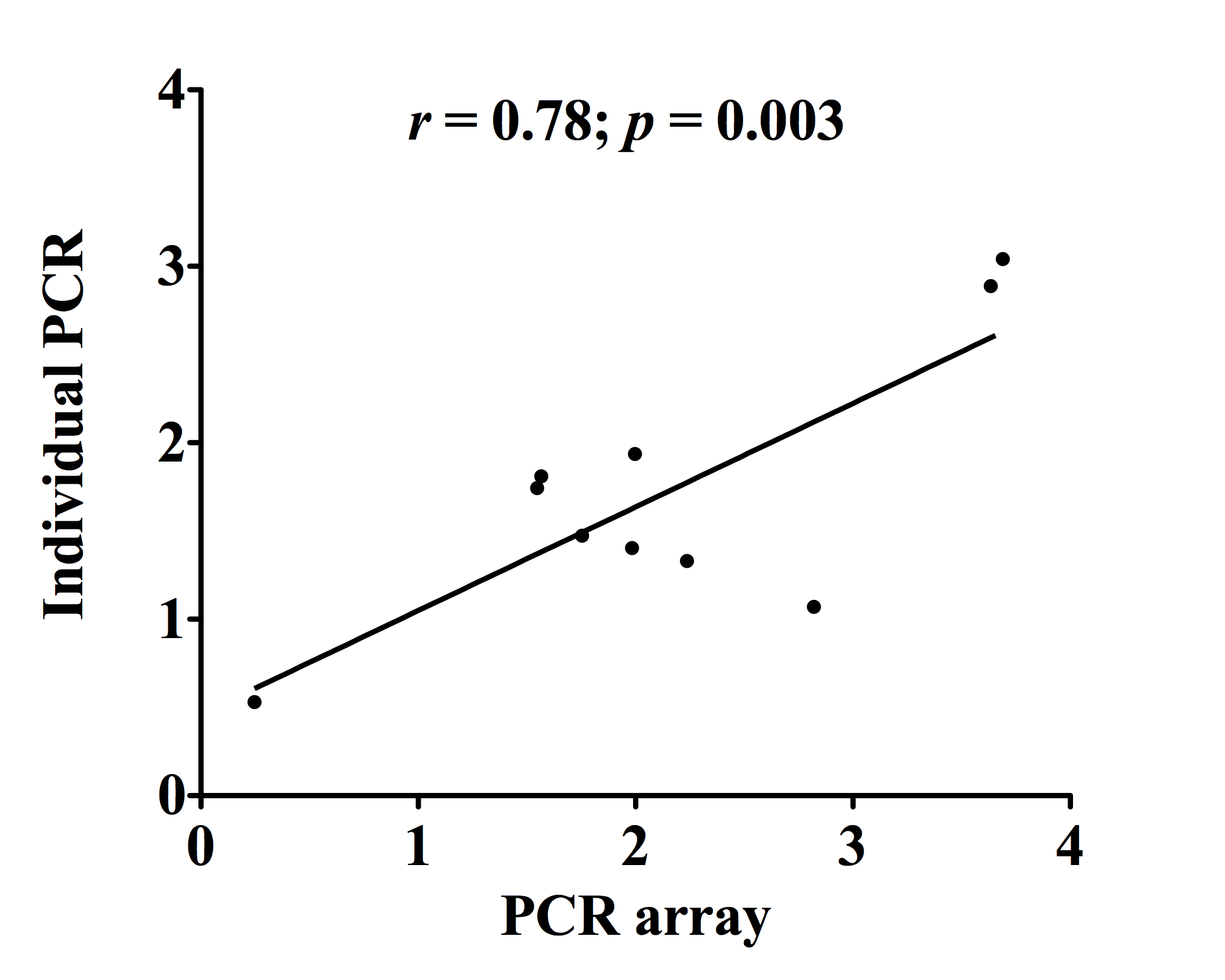

Supplement: Figure S3 — Individual PCR validation of a selected set of deregulated miRNAs in L. major -infected human macrophages. Scatter plot analysis shows correlation between mean expression levels of nine miRNAs measured by array analysis (PCR array) and mean expression levels tested using individual qRT-PCR (Individual PCR) in three donors. Correlation coefficient r and statistical p values are indicated. Results were expressed using the 2−ΔΔCt method. (TIFF) [file pntd.0002478.s003.tiff]

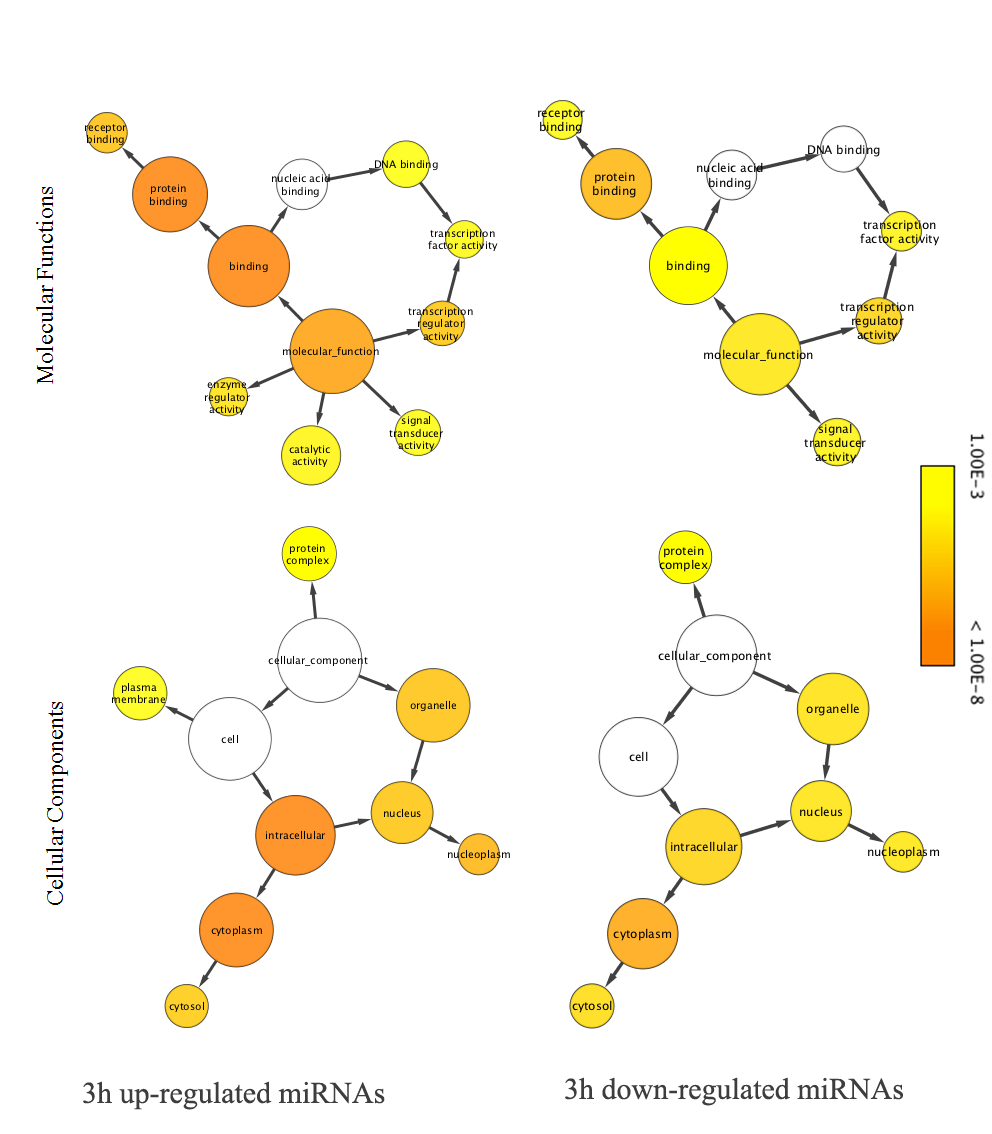

Supplement: Figure S4 — Molecular functions and cellular components of L. major -infected human primary macrophage miRNA-targets at 3 h post-infection. Regulatory network was obtained after GO enrichment deduced from analysis of up- or down-regulated miRNA-targets. Yellow color gradient intensity correlates with up- or down-regulation levels. White nodes are not significantly overrepresented. The area of each node is proportional to the number of genes in the set annotated to the corresponding GO category. Interactions were visualized as a network using Cytoscape and BINGO plugin. (TIF) [file pntd.0002478.s004.tif]

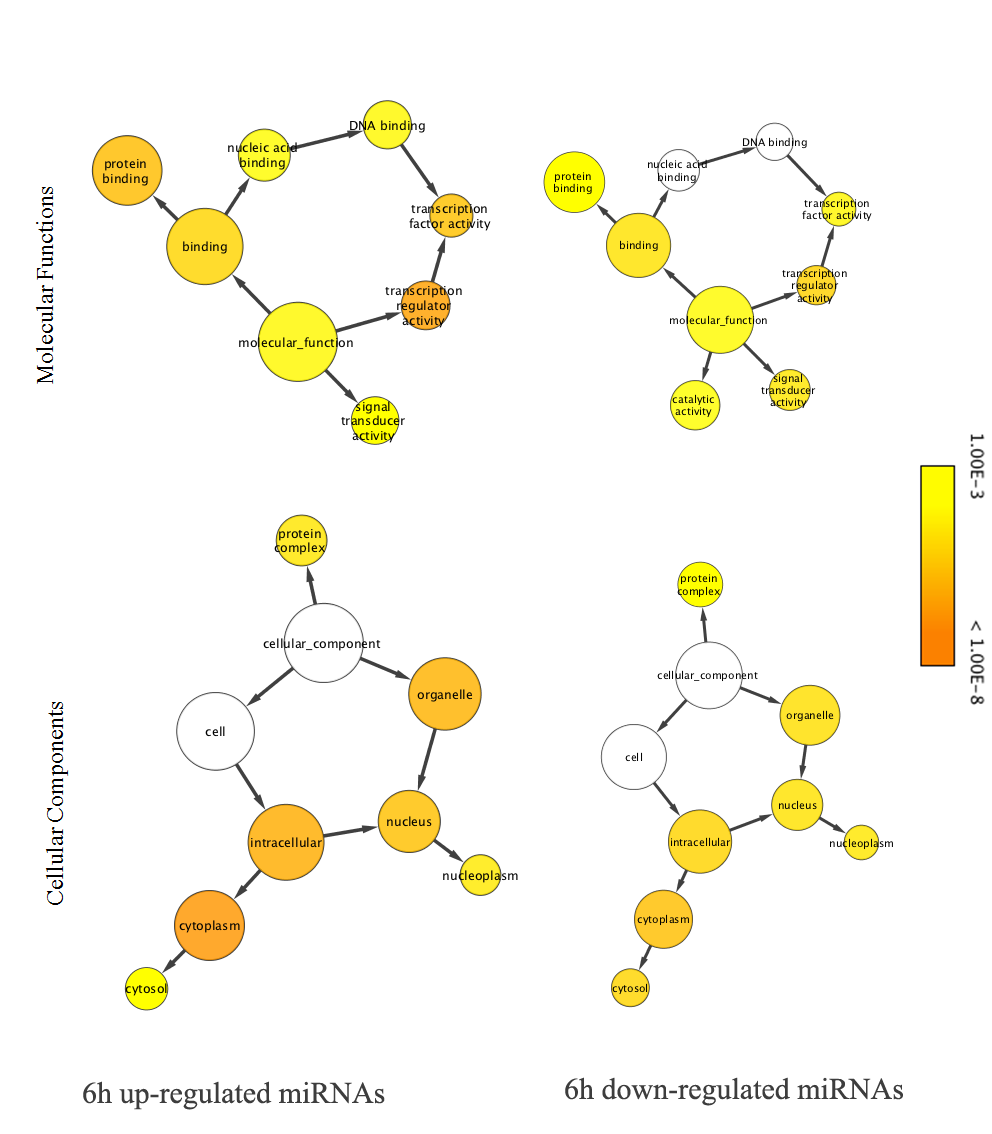

Supplement: Figure S5 — Molecular functions and cellular components of L. major -infected human primary macrophage miRNA-targets at 6 h post-infection. Regulatory network was obtained after GO enrichment deduced from analysis of up- or down-regulated miRNA-targets. Yellow color gradient intensity correlates with up- or down-regulation levels. White nodes are not significantly overrepresented. The area of each node is proportional to the number of genes in the set annotated to the corresponding GO category. Interactions were visualized as a network using Cytoscape and BINGO plugin. (TIF) [file pntd.0002478.s005.tif]

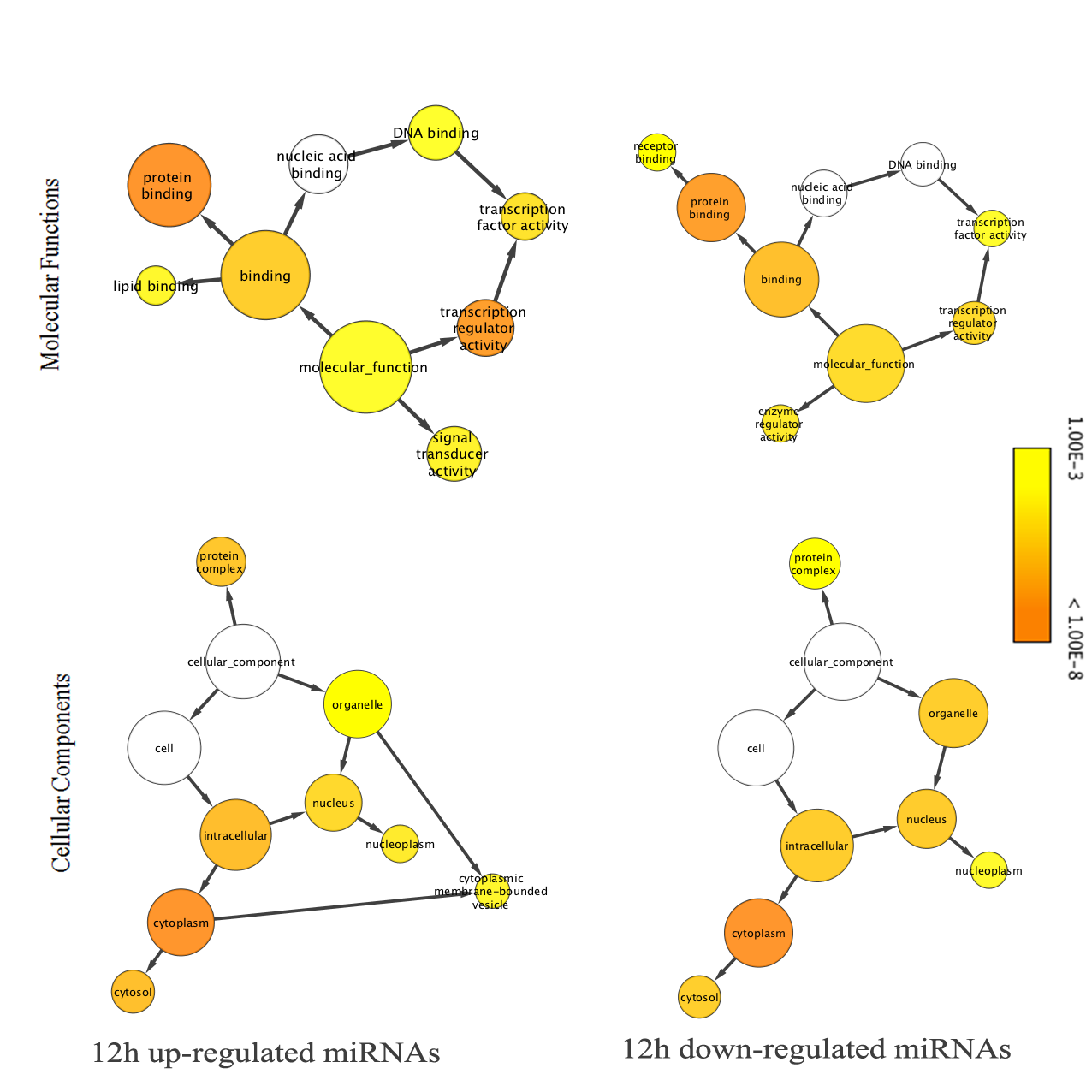

Supplement: Figure S6 — Molecular functions and cellular components of L. major -infected human primary macrophage miRNA-targets at 12 h post-infection. Regulatory network was obtained after GO enrichment deduced from analysis of up- or down-regulated miRNA-targets. Yellow color gradient intensity correlates with up- or down-regulation levels. White nodes are not significantly overrepresented. The area of each node is proportional to the number of genes in the set annotated to the corresponding GO category. Interactions were visualized as a network using Cytoscape and BINGO plugin. (TIF) [file pntd.0002478.s006.tif]

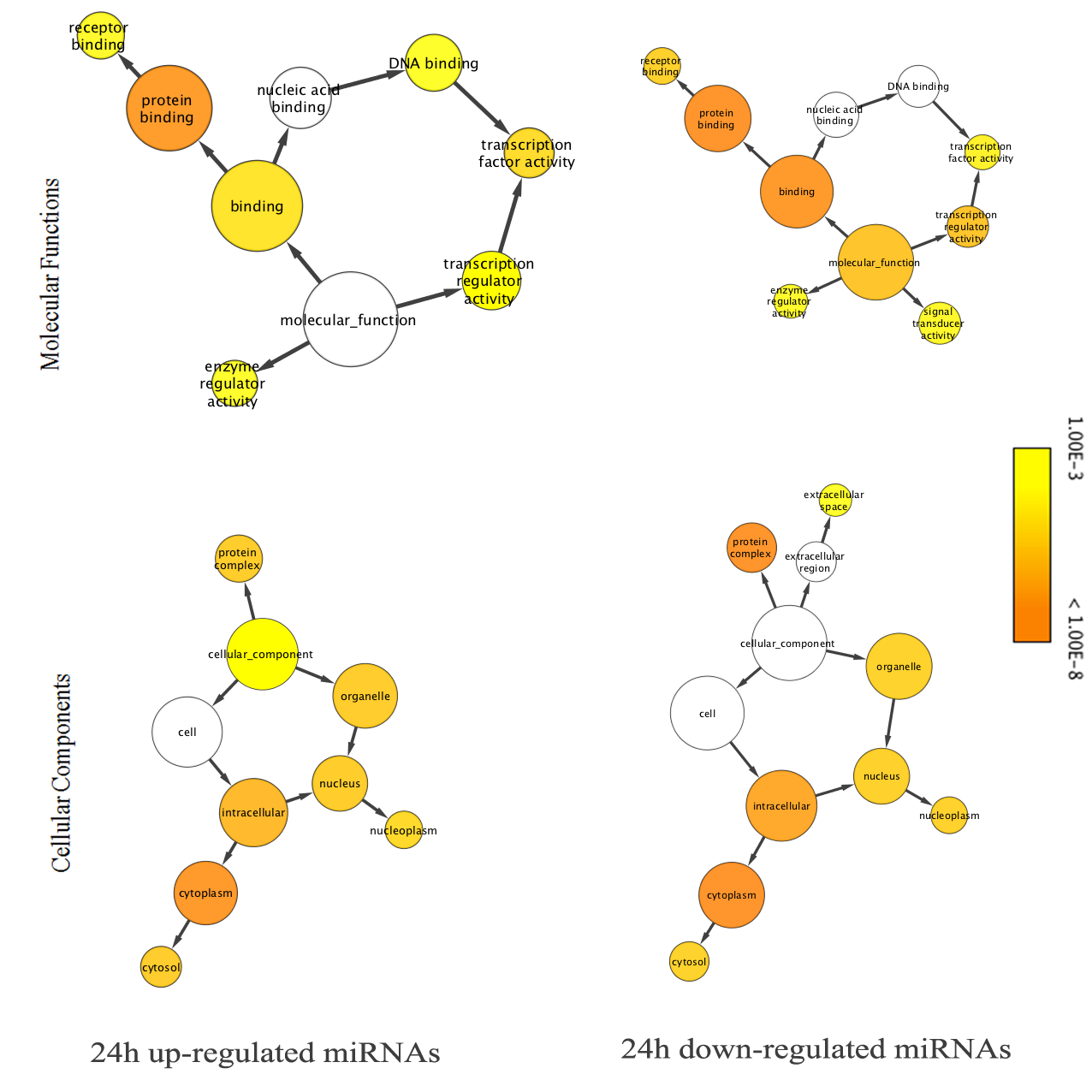

Supplement: Figure S7 — Molecular functions and cellular components of L. major -infected human primary macrophage miRNA-targets at 24 h post-infection. Regulatory network was obtained after GO enrichment deduced from analysis of up- or down-regulated miRNA-targets. Yellow color gradient intensity correlates with up- or down-regulation levels. White nodes are not significantly overrepresented. The area of each node is proportional to the number of genes in the set annotated to the corresponding GO category. Interactions were visualized as a network using Cytoscape and BINGO plugin. (TIF) [file pntd.0002478.s007.tif]

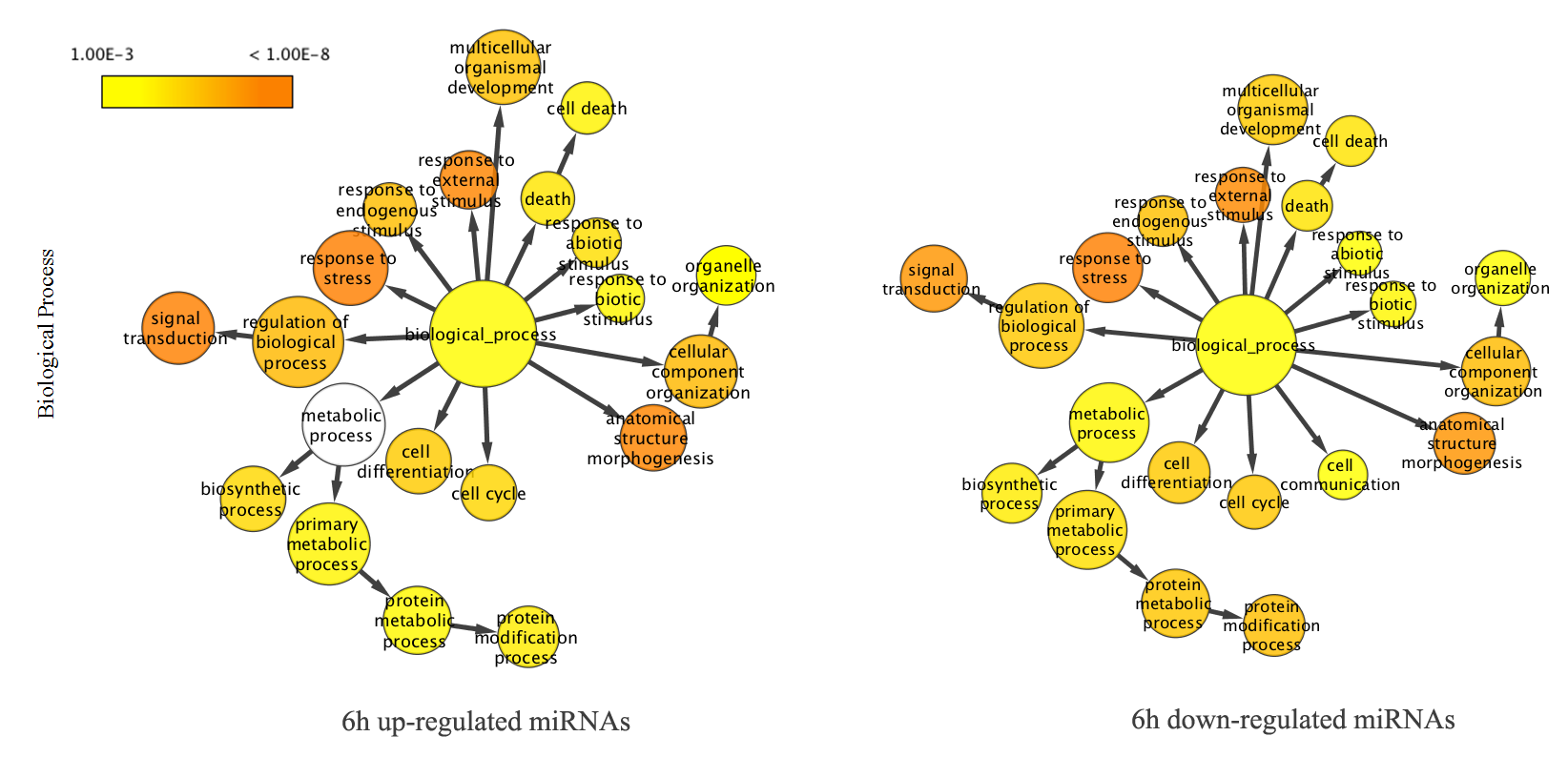

Supplement: Figure S8 — Biological processes deduced from analysis of deregulated miRNA-targets in L. major -infected human macrophages at 6 h post-infection. Yellow color gradient intensity correlates with up- or down-regulation levels. White nodes are not significantly overrepresented. The area of each node is proportional to the number of genes in the set annotated to the corresponding GO category. Interactions were visualized as a network using Cytoscape and BINGO plugin. (TIF) [file pntd.0002478.s008.tif]

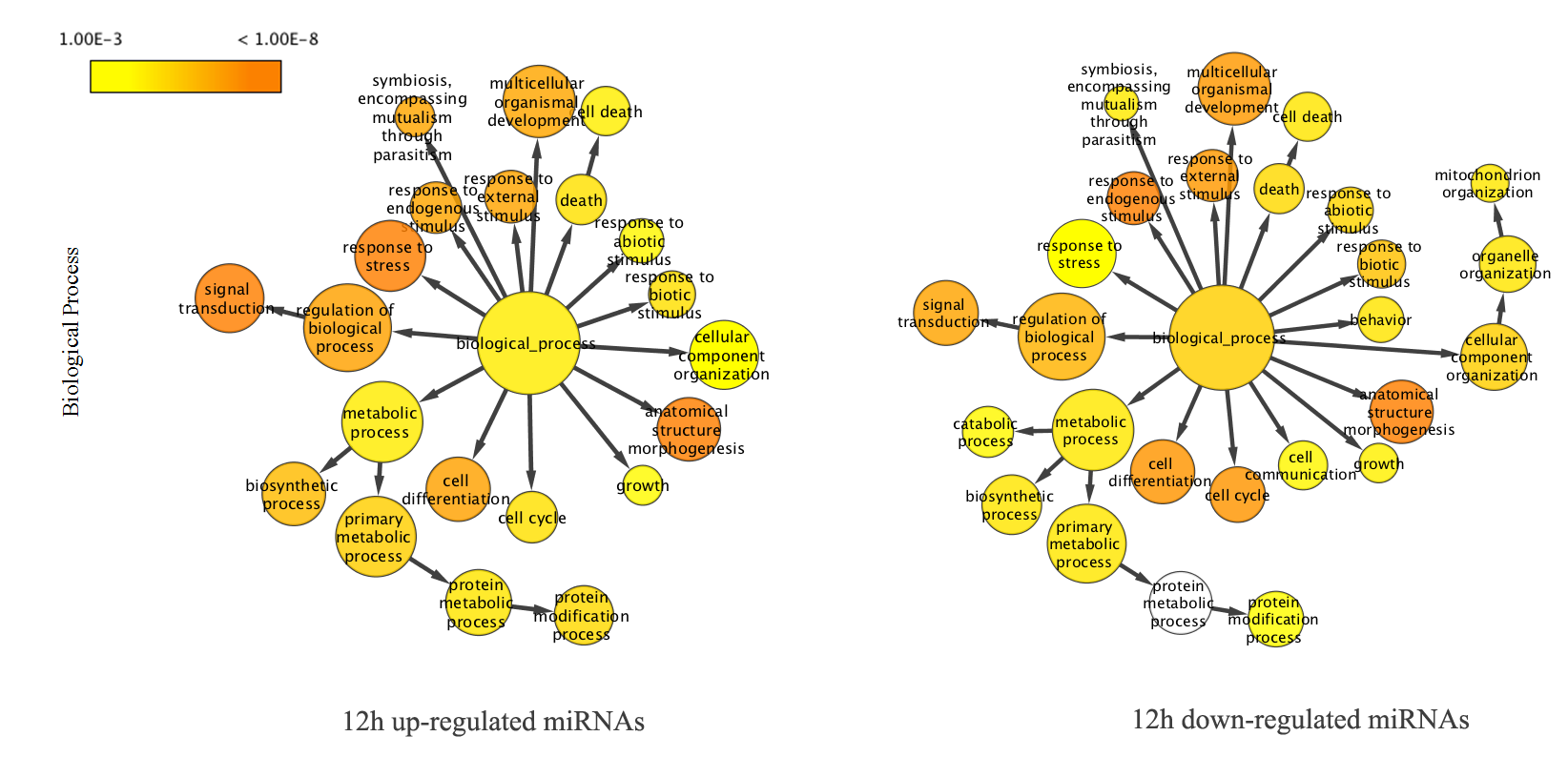

Supplement: Figure S9 — Biological processes deduced from analysis of deregulated miRNA-targets in L. major -infected human macrophages at 12 h post-infection. Yellow color gradient intensity correlates with up- or down-regulation levels. White nodes are not significantly overrepresented. The area of each node is proportional to the number of genes in the set annotated to the corresponding GO category. Interactions were visualized as a network using Cytoscape and BINGO plugin. (TIF) [file pntd.0002478.s009.tif]

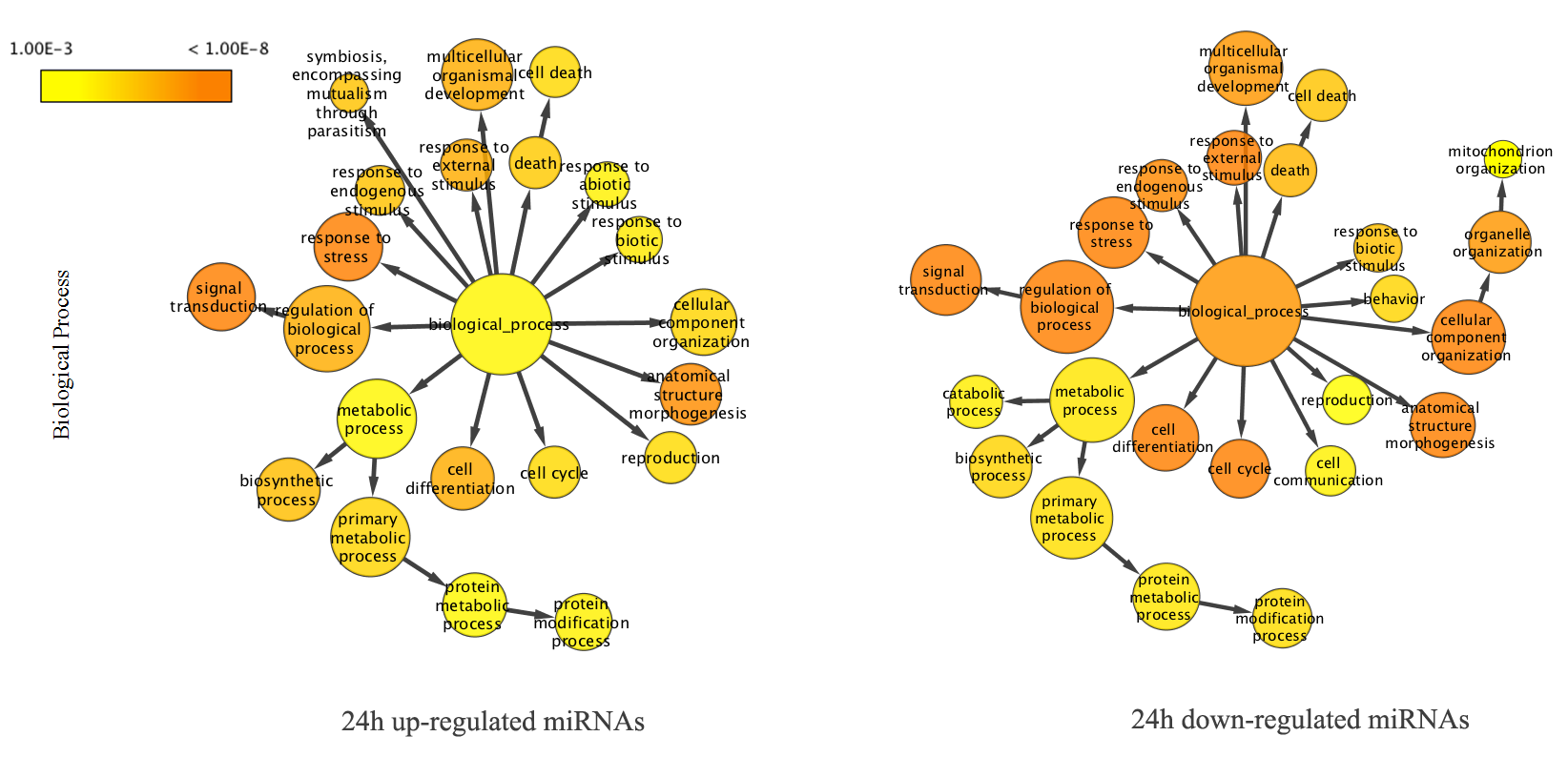

Supplement: Figure S10 — Biological processes deduced from analysis of deregulated miRNA-targets in L. major -infected human macrophages at 24 h post-infection. Yellow color gradient intensity correlates with up- or down-regulation levels. White nodes are not significantly overrepresented. The area of each node is proportional to the number of genes in the set annotated to the corresponding GO category. Interactions were visualized as a network using Cytoscape and BINGO plugin. (TIF) [file pntd.0002478.s010.tif]

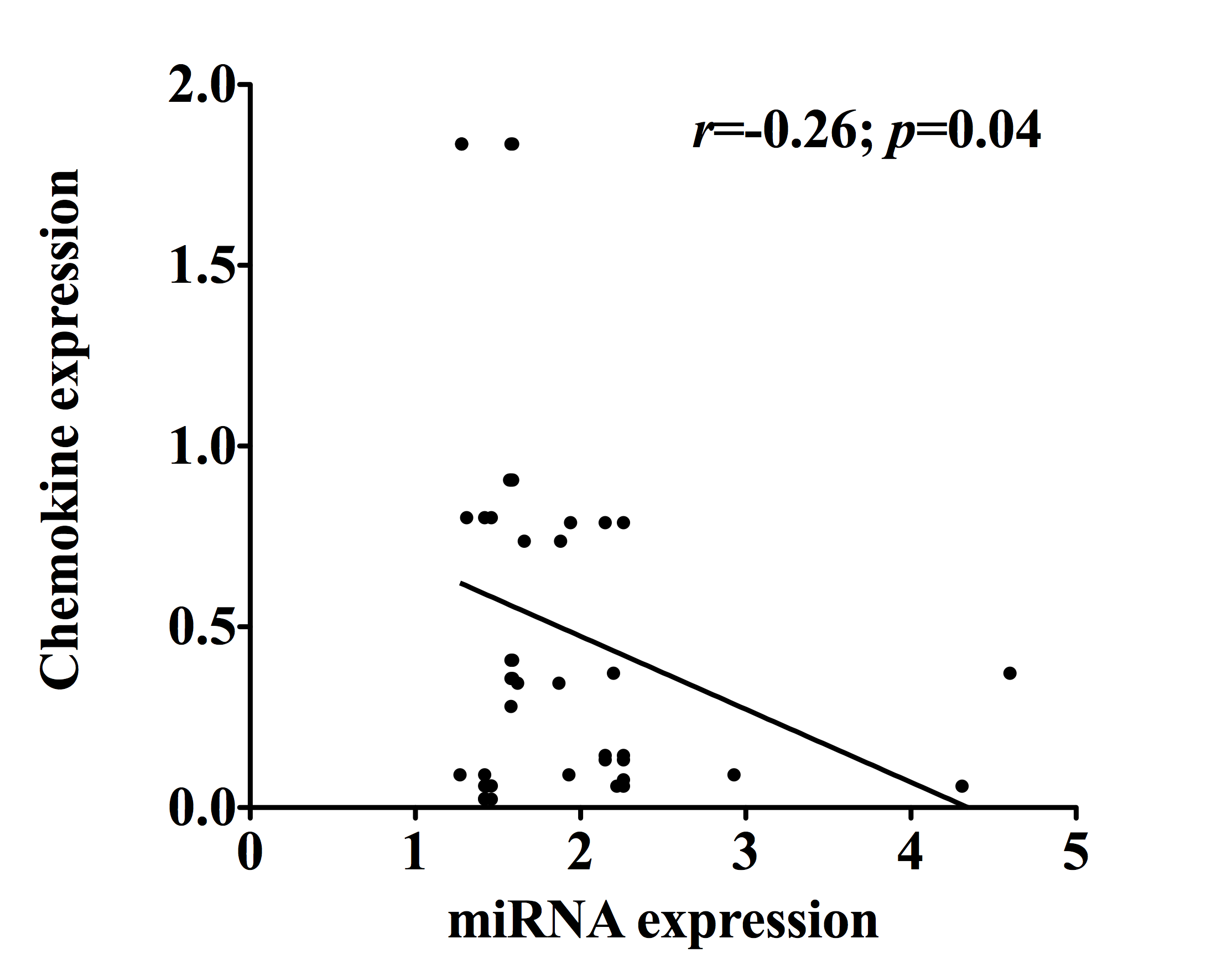

Supplement: Figure S11 — Scatter plot analysis showing a negative correlation between expressions of an up-regulated set of miRNAs and their targeted chemokine transcripts. Expression of let-7a, miR-25, miR-26a, miR-140, miR-146a and miR-155 at 3 h and miR-23b and miR-132 at 6 h post-infection of three healthy donors (D1, D2 and D3) is negatively correlated with CCL2, CCL5, CXCL10, CXCL11 and CXCL12 mRNA levels at 12 and 24 h post-infection in L. major-infected human macrophages. Correlation coefficient r and statistical p values are indicated. Results were expressed using the 2−ΔΔCt method. (TIFF) [file pntd.0002478.s011.tif]
